# A tailored, electronic textile conformable suit for large-scale spatiotemporal physiological sensing in vivo

**DOI:** 10.1038/s41528-020-0068-y

**Published:** 2020-04-23

**Authors:** Irmandy Wicaksono, Carson I. Tucker, Tao Sun, Cesar A. Guerrero, Clare Liu, Wesley M. Woo, Eric J. Pence, Canan Dagdeviren

**Affiliations:** 1 Media Lab, Massachusetts Institute of Technology, Cambridge, 02139 MA USA; 2 Mechanical Engineering, Massachusetts Institute of Technology, Cambridge, 02139 MA USA; 3 Electrical Engineering and Computer Science, Massachusetts Institute of Technology, Cambridge, 02142 MA USA; 4 Architecture and Planning, Massachusetts Institute of Technology, Cambridge, 02139 MA USA

**Keywords:** Sensors and probes, Electrical and electronic engineering

## Abstract

The rapid advancement of electronic devices and fabrication technologies has further promoted the field of wearables and smart textiles. However, most of the current efforts in textile electronics focus on a single modality and cover a small area. Here, we have developed a tailored, electronic textile conformable suit (E-TeCS) to perform large-scale, multimodal physiological (temperature, heart rate, and respiration) sensing in vivo. This platform can be customized for various forms, sizes and functions using standard, accessible and high-throughput textile manufacturing and garment patterning techniques. Similar to a compression shirt, the soft and stretchable nature of the tailored E-TeCS allows intimate contact between electronics and the skin with a pressure value of around ~25 mmHg, allowing for physical comfort and improved precision of sensor readings on skin. The E-TeCS can detect skin temperature with an accuracy of 0.1 °C and a precision of 0.01 °C, as well as heart rate and respiration with a precision of 0.0012 m/s^2^ through mechano-acoustic inertial sensing. The knit textile electronics can be stretched up to 30% under 1000 cycles of stretching without significant degradation in mechanical and electrical performance. Experimental and theoretical investigations are conducted for each sensor modality along with performing the robustness of sensor-interconnects, washability, and breathability of the suit. Collective results suggest that our E-TeCS can simultaneously and wirelessly monitor 30 skin temperature nodes across the human body over an area of 1500 cm^2^, during seismocardiac events and respiration, as well as physical activity through inertial dynamics.

## Introduction

In recent years, we have witnessed a vast advancement towards flexible and stretchable devices^1,2^. The current form-factor of medical devices that are rigid and boxy starts to become soft and conformable^3,4^. This brings out health monitoring that is non-obtrusive, imperceptible, and closer to our body, even when we are away from the hospital^5^. There are two major classes of wearable electronics for healthcare: on-skin, and textile electronics. Thin, soft and skin-like electronics in the form of a patch, with wireless capabilities, have been developed to precisely detect various physiological signals from the human body, such as electrophysiology^6^, temperature^7^, pulse oximetry^8^, blood pressure^9^, hydration^10^, and others^11^. They are made either by designing a particular structure that can withstand strain on a deformable polymeric substrate, or by using intrinsically stretchable materials^12^. On the other hand, textiles and clothing are ubiquitous in our daily life. We wear and wash them regularly, and they give us comfort and protection from the outside environments. Being the closest layer to our body, they provide an ideal platform for the integration of electronics to monitor physiological processes through the skin. Electronic devices integrated into textiles can, therefore, offer several advantages, such as enhanced mobility and comfort for the user^13^. Textile also serves an excellent substrate for sensing throughout dynamic activities and environments, where robustness and washability are critical as the substrate undergoes multiple stretching, friction, and is frequently exposed to dirt and humidity. Several efforts have been conducted to integrate electronics into textiles, for instance, by coating yarns with metal or printing conductive inks on fabrics to serve as electrodes for electrophysiology^14,15^, sewing and attaching functional threads and fabrics^16^, weaving electronics fabricated on polyimide strips for humidity^17^, temperature^17^, pulse oximetry^18^, and gas^19^ sensing, as well as developing electronic fibers for seamless woven electronic textiles^20^. Some of these intelligent textiles, however, are not scalable for large-area sensing and do not allow stretchability for the application of skin-contact sensing for electronic suits.

It is also worthy to note that current on-skin and wearable devices mostly measure a single parameter at a particular location of the body^21^. Distributed sensor networks that can spatiotemporally map multiple physiological processes and physical movements in different regions of the body (Supplementary Table 1) are a valuable tool for clinicians, as they can provide a rich dataset to assess a health condition, predict disease, or advance sports science and analytics^22,23^. A specific example is soft, battery-free epidermal sensors that can be adhered to various regions of the body to perform full-body skin temperature and physical pressure mapping^24^. These sticker-like sensors are used in sleep studies to help with the treatment of sleep disorders, jet lag, and pressure ulcers on a clinical bed setup. Distributed skin temperature mapping has also been demonstrated to study thermoregulation efficiency in athletic performance^25^, as well as dermatome abnormality through regional nerve root damage^26^. However, even though they are wireless, these epidermal sensors require a Near Field Communication (NFC) reader around the vicinity to power the electronics and collect the data. They would be also challenging to be used while performing dynamic activities, which limit its applications outside the bed. Their soft, fragile nature and adhesive tape application on the skin may restrain them from long-term operations. Other wireless on-skin devices are integrated with batteries^27,28^. However, having multiple devices with their independent power sources tend to be cumbersome when one needs to replace and charge every single device, rather than wearing a centralized, system-on-textile garment that could perform all of the functions. Several on-skin and textile electronic devices also require specific materials and microfabrication techniques to be developed, resulting in relatively high cost and effort for mass manufacturing and large-scale deployment^29,30^.

Recent work also focus on the design of customizable, modular, and reconfigurable soft electronics, but not so many apply these design principles to textile-based applications^31–33^. The wide variations in human body size and shape prove to be a challenge on the design and development of smart clothing. Accordingly, a universal platform of sensor networks on textiles as well as their hardware-software integration, must be established^34^. With this standardization, industries can, therefore, work on specific parts, such as sensor modules and not be concerned about designing a full wearable system. Further processes can then focus on personalization of smart clothing based on the user’s requirements and needs^35^.

Through this work, we have developed a technique of combining thin, customizable conformable electronic devices, including interconnect lines and off-the-shelf integrated circuits, with plastic substrates that can be woven into knitted textile using an accessible and high-throughput manufacturing approach. Similar to a compression garment, the nature of this stretchable knitted textile will allow intimate contact between electronics and the skin^36^. Our technique creates a platform to integrate a large assortment of conformable electronic components in a suit for large-scale physiological and physical activity sensing on the body. We demonstrate the capability of our electronic textile conformable suit (E-TeCS) for distributed, wireless physiological sensing, such as temperature, respiration and heart-rate detection, and physical activity monitoring around the human body during a physical exercise. Repeated mechanical cycling tests also prove the durability of the knitted textile for daily wear.

## Results

### System overview

**Fig. 1:**
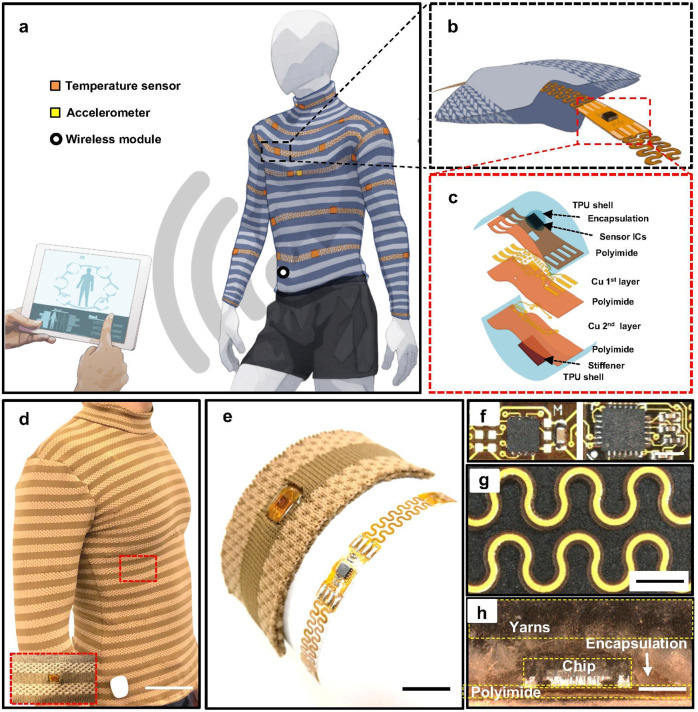
A tailored, electronic textile conformable suit (E-TeCS) for distributed sensing wirelessly. Illustration of (**a**) spatiotemporal sensor mapping of the body with temperature and accelerometer (heart beat and respiration), (**b**) textile channel for embedding flexible-stretchable electronic strips, and (**c**) exploded view of a sensor island. A photograph of final E-TeCS prototype (**d**) showing its conformability to the wearer (scale bar: 10 cm), (**e**) bare flexible-stretchable electronic strip (right) and woven electronic strip in a knit textile (left) (scale bar: 1 cm). Microscopy image of a (**f**) temperature (left), accelerometer (right, scale bar: 3 mm), and (**g**) interconnect modules (scale bar: 2 mm), and (**h**) cross-sectional view of an E-TeCS module embedded in a polydimethylsiloxane (PDMS) layer (scale bar: 2 mm).

Figure 1a illustrates the concept of an E-TeCS that monitors the human skin surface temperature distribution, heart rate, and respiration. The suit is tailored from a customized fabric that can be integrated with an assortment of sensor integrated circuits (ICs) and interconnects in the form of flexible-stretchable electronic strips. The textile platform consists of channels or pockets for the weaving of these electronic strips (Fig. 1b). The sensor ICs and interconnects are developed using two-layer industrial flexible printed circuit board (PCB) processes (Fig. 1c, 4.1a and see Methods), with additional steps for chip and passive component assembly and encapsulation with thermoplastic polyurethane (TPU) (TE-11C, Dupont) and washable encapsulant (PE773, Dupont).

The tailored approach through body fitting results in E-TeCS that fully conforms to the curvature of the body (Fig. 1d). The textile channels for embedding the electronics further enhance the comfort of the suit. Figure 1e presents a photograph of a temperature device (MAX30205, Maxim Integrated) and the device woven into the customized textile. Figure 1f shows the sensor island for temperature (right) and accelerometer (left) respectively, with an outline size of 0.6 cm × 1 cm. The two layers, that consist of serpentine interconnects (Fig. 1g) of 18 µm thick and 300 µm track width of cupper (Cu) and are sandwiched between 75 µm thick and 700 µm track width of polyimide (PI), serve as the bridge for a total of four bus lines as Inter-integrated Communication (I^2^C) network architecture. From the cross-sectional microscope image of the device woven into the customized textile (Fig. 1h), it can be seen that there are four main layers: the textile, encapsulation, electronic chip, and polyimide (PI).

### Modular sensor networks

As shown in Supplementary Fig. 1a, we designed and fabricated seven different modules: four temperature sensing modules, one inertial sensing module, and two interconnection modules. In an area of 25 cm × 27.5 cm flexible board (FPCB, KingCredie), we can fit a total of 66 temperature sensors and 20 interconnection strips, demonstrating the large-amount, rapid manufacturability of this approach. The temperature sensor (MAX30205, Maxim Integrated) has an accuracy of 0.1 °C between 37–39 °C, and a 0.0039 °C resolution, which we rounded up programmatically to 0.01 °C. This sensor can have up to 32 unique addresses, which can be set by connecting ground (GND), power supply (VDD), data line (SDA) or clock line (SCL) signal to the A0, A1, and A2 pins on the chip. Given that there are eight combinations possible in these three pins for each signal, we designed four different hard-wired A0, A1, and A2 pins (Supplementary Fig. 1b) to voltage supply (VDD) or ground (GND) and data-line (SDA) or clock-line (SCL), represented by M, M1, M2, and M3. Each temperature module can be manually joined by soldering the jumpers (Supplementary Fig. 1c), in order to access all of the possible 32 addresses. The capacitor complement of the MAX3025 is used as a decoupling capacitor to stabilize the local VDD supply from high-frequency noise and voltage ripples. The mechano-acoustic sensor or inertial measurement unit (IMU) (MPU6050, InvenSense) is capable of measuring 3-axis gyroscope and 3-axis accelerometer, with a programmable accelerometer range of ±2 g to 16 g, the highest precision of 0.00012 g or 0.0012 m/s^2^, and a maximum of two addresses in one I^2^C bus. We designed four pads at each side of all sensor modules for connection to power and signal lines (VDD, SCL, SDA, GND).

The sensing modules or islands can be joined to the interconnect modules and each other by soldering the four pads together (Supplementary Fig. 1d). The interconnect strips have multiple islands of pads with an area of 1 mm × 4 mm in between serpentine interconnects (Supplementary Fig. 1e). The pad design enables the interconnect strips to be reconfigurable. It can be cut and joined to any length needed for connection to the sensor islands. The female headers or holes at the end of these interconnect strips can be used for textile-hardware connections by looping conductive threads or thin wires.

All of the sensor modules can be connected to the main module for powering, processing, and wireless communication through the I^2^C bus interface with four signal wires (VDD, SCL, SDA, GND). For a single I^2^C bus, the maximum sensor nodes it can access is 2^8^ = 128 addresses. This means that the system can handle up to 32 temperature sensors (0×40 to 0×5f in 7-bit address) and 2 inertial measurement units (IMU) (0×68 and 0×69) with minimal wirings. The corresponding address of the temperature sensors is given in Supplementary Table 2. Since every sensor module in our system have its own reading and processing happen locally, adding several sensor nodes of different nature will not introduce cross-talk, as long as they have unique sensor address.

Supplementary Fig. 2a illustrates the concept of a modular sensor network architecture embedded in a piece of fabric. Each sensor can be connected to each other with the interconnects in a horizontal manner, where the signal gets collected by the external layer, which consists of a Bluetooth low-energy (BLE) module, a microprocessor, and a power source. We developed a prototype (Figure S2b) to demonstrate the scalability of the sensor-integrated fabric, as shown in Supplementary Fig. 2b–d. As more fabrics and sensors are joined, I^2^C address scanning from a micro-controller showed an increase of the number of sensor addresses detected (Supplementary Fig. 2e–g). This demonstration reflects the possibility of roll-to-roll manufacturing of sensor-embedded fabrics that can be cut in any size, joined, and tailored for various needs and applications.

### Temperature and inertial sensor characterization

**Fig. 2:**
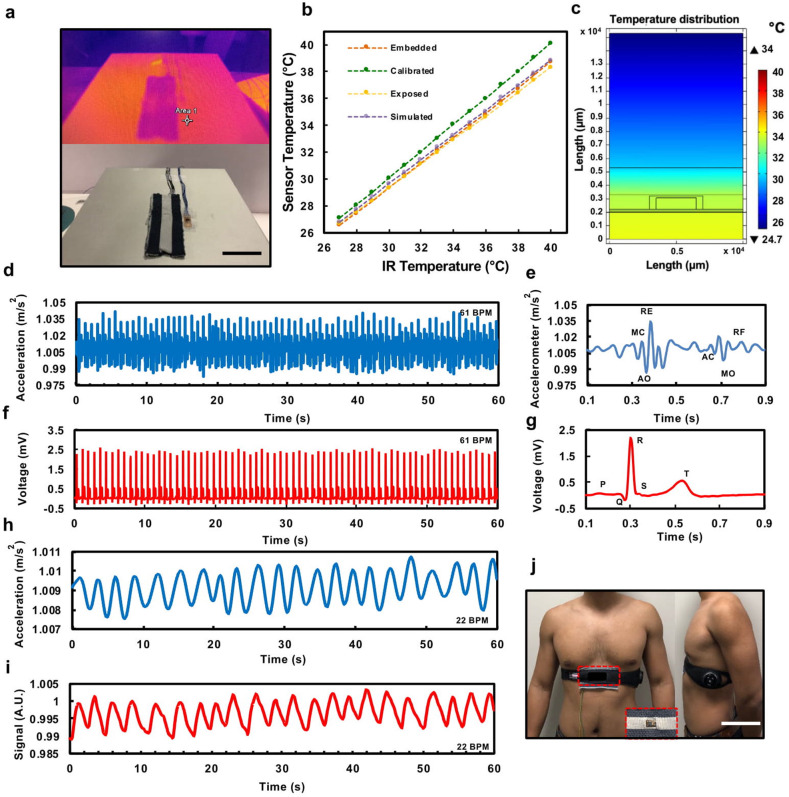
Thermal characterization of temperature sensor-embedded fabric. The photography of (**a**) the hot-plate setup and thermal image between bare temperature sensor and the one integrated in a fabric (scale bar: 3 cm). **b** Characterization, simulation, and calibration results of the IR camera thermal test. **c** FEM thermal distribution for a source temperature of 34 °C. Simultaneous measurement of accelerometer SCG with a commercial ECG. **d** Mechano-acoustic response of accelerometer embedded in a fabric for 1 min. A.U.: arbitrary unit. **e** Magnified view of heart acoustic signals in (**d**), MC, mitral valve closure; AO, aortic valve opening; RE, rapid ventricular ejection; AC, aortic valve closure; MO, mitral valve opening; RF, rapid ventricular filling. **f** A commercial ECG response under 1 min. **g** Magnified view of the ECG response in (**f**). **h** Commercial accelerometer and (**i**) Zephyr Biopatch respiratory waveform. A.U.: arbitrary unit, and (**j**) Zephyr Biopatch and fabric accelerometer sensor placement (scale bar: 10 cm).

We performed infrared (IR) thermography cross-validation with a total of four trials (*n* = 4), of an encapsulated device without any integration to a fabric and an encapsulated device embedded in a fabric channel (Fig. 2a). See Methods for further description of the experimental setup. The results in Fig. 2b show that there is an increasing offset as the temperature rises in both cases, with the fabric device exhibiting better performance or higher temperature; this is due to the insulating behavior of the fabric layer that keeps the temperature from distributing to the environment. These values in Supplementary Fig. 3a were consistent with those determined by a two-dimensional (2-D) finite element model (FEM) simulation in Supplementary Fig. 3b–g. Based on the FEM simulation and experimental results, the sensor required a calibration factor, defined by an offset and a multiplier from linear fitting that converts the sensor reading close to the temperature obtained by the IR camera (Supplementary Fig. 3a).

2-D FEM model was created in accordance with the structure of the sensor embedded into the textile, to study the temperature distribution across the cross-section of the sensor. The heat is transferred from the heat source (Digital Hotplate, Torrey Pines Scientific) to the bottom surface of the packaged sensor due to the thermal contact, and ultimately transmitted from the top textile layer to the external environment, primarily in the form of convection and radiation. We simulated no airflow, even though the air is considered in the external environment. The ambient temperature was specified as 24.85 °C, similar to the ambient temperature at the time of experimental characterization. The steady-state temperature distribution was then theoretically simulated. The simulated results matched the experimental results with a tolerance of 0.2308 ± 0.0488 °C. Figure 2c shows a sample of the simulated thermal distribution across the 2-D model when the hot-plate temperature is 34 °C, while Supplementary Fig. 3b–g shows the distribution when the hot-plate temperature is ranged from 30 to 40 °C.

Seismocardiography (SCG) records the subtle motions around the body due to the atrial muscle contractions and blood ejection as the heart pumps. The frequency characteristic waveform of SCG thus reflects cardiac mechanical events. It can be unobtrusively monitored by attaching IMUs to the body or integrating them to objects that will physically touch the body^37,38^. Depending on the location of the IMUs, they can also capture body motions caused by the contraction and dilation of the lungs, which relate to the breathing mechanism. We placed an IMU right below the sternum as it has been shown to be the most sensitive location to detect both heart and breathing activities^39,40^. We assembled, encapsulated, and integrated an accelerometer module with a customized fabric patch. Figure 2j shows our mechano-acoustic element embedded in a fabric and placed right below the sternum, with a commercial electrocardiography (ECG) and respiration (Zephyr BioPatch, Medtronic) strap as the cross-validation device for simultaneous measurement of SCG and ECG.

A single cardiac cycle represents the contraction (systole) and relaxation (diastole) of heart muscle motions of the atrium and ventricular chamber. These motions induce electrical activities, which are followed by mechanical movements as the heart chambers contract and the valves close. These electromechanical coupling features are imperative in ECG and heart auscultation. Figure 2d, f show ECG and SCG signals measured simultaneously from a healthy male subject (age 25). The SCG data are given by the accelerometer (MPU-6050, InvenSense) z-axis value, with a sensitivity setting of 2 g, precision of 0.0012 m/s^2^, and a sampling frequency of 100 Hz. A finite impulse response (FIR) low-pass filter is used (see Methods) to process the raw data (Supplementary Fig. 4) eliminating respiratory waveforms. Magnified views of a single cardiac cycle (Fig. 2e, g) highlight all the critical features of these two waveforms, such as the mitral valve closure (MC), aortic valve opening (AO), and rapid ventricular ejection (RE) occurring right after R-peak or ventricle depolarization and aortic valve closure (AC), mitral valve opening (MO), and rapid ventricular filling (RF) after T-peak, and ventricle relaxations^39^.

From the raw data in Supplementary Fig. 4, not only we could collect SCG data that provide information on the heart activity, but we could also find insights on the breathing activity due to the lung and diaphragm mechanical movements. For respiratory waveform, FIR low-pass filter is also used to eliminate high-frequency signals due to heart-beat events and obtain the direct current (DC) component of the signals. The result shows a breathing waveform (Fig. 2h) that exhibits a similar response in comparison to a commercial device (Zephyr BioPatch, Medtronic) as shown in Fig. 2i.

### Development of personalized E-TeCS

**Fig. 3:**
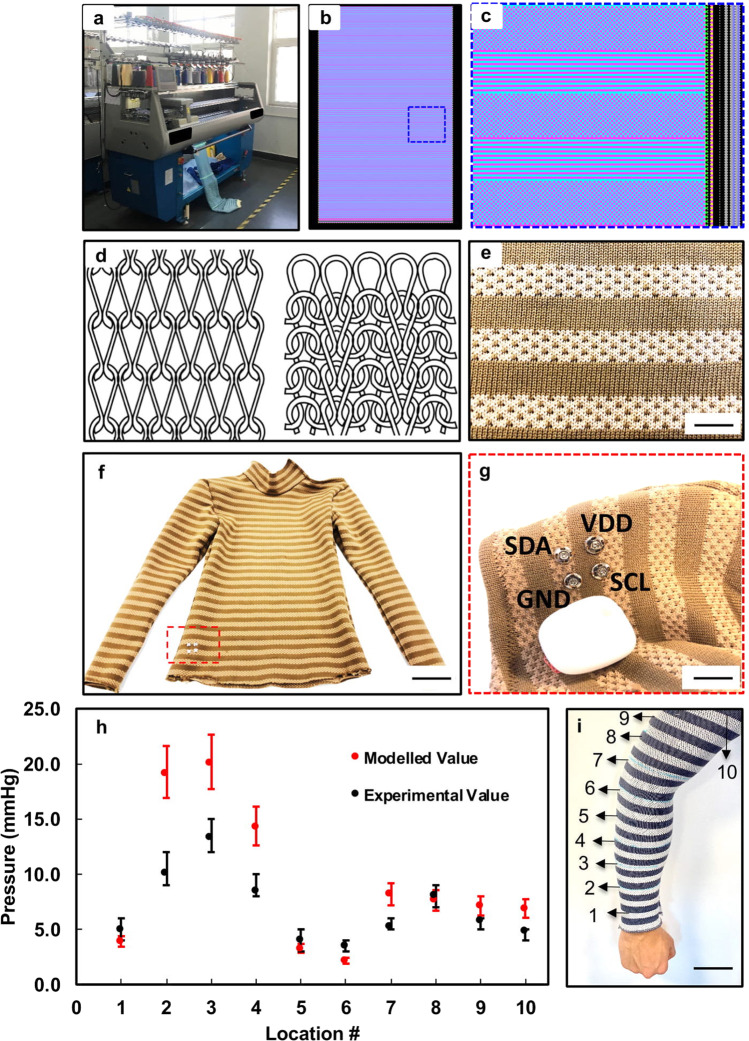
Customized fabrics through digital knitting. Photography of a (**a**) two-bed knitting machine (scale bar: 50 cm). **b** Screen capture of digital knitting software interface. **c** The structure of the customized fabrics in visual programming, the stripes correspond to hollow two-layer fabrics, and the checkered pattern represents interlocking mechanism. **d** Sketch of a single jersey knit loop structure (left) and interlocking loop structure (right). **e** A photograph of the E-TeCS fabric channels (scale bar: 1 cm). **f** Final prototype image of a E-TeCS (scale bar: 8 cm) with (**g**) exploded view of the detachable main processing and communication module (scale bar: 2 cm), **h** Experimental and modeled value of the compression pressure across the circumferences of the arm, as illustrated in (**i**). **i** A photograph of compression test for E-TeCS in ten different locations (scale bar: 5 cm).

Digital knitting is a programmable, automatic machine process (Fig. 3a) of stitching interlocked loops from multiple strands of yarn^41^. It uses several needles or hooks to arrange the interlocking mechanism of loops into fabrics. The process of knitting starts with multiple cones of yarn that gets pulled into the machine by yarn carriers until a certain pre-programmed tension is achieved. The carriers then slide back-and-forth horizontally while the needles catch the yarns to form the loops. Each carrier can be sequentially controlled to slide and combine different yarns to form structural or color patterns. The programming interface consists of two grid sections (Fig. 3b, c). The left grid is used to develop the shape and pattern of the knit fabrics through *x–y* color block programming, where each color and logo represent specific knit operation.

Using a flat two-bed digital knitting machine (Super-J 212, Matsuya), we patterned textile channels using a combination of two-layer jersey (left) and interlocked knitting (right), as illustrated in Fig. 3d. Figure 3e shows a region of the resultant fabric, with four textile channels and three interlocked stripes. The single-color stripes in this piece consist of two layers of separated fabric, while the dotted stripes represent the interlocked patterns, which combined two fabric layers into one. We digitally knitted three fabrics with a size of 55 × 120 cm: one for front-side, one for back-side, and one for a pair of long-sleeve. The channel design of our digitally knit fabric was done based on the size of our sensing and interconnect modules. As shown in Supplementary Fig. 1a, the width of our sensing and interconnect modules are 0.6 cm. Therefore, we decided to design 1 cm channels on our digitally knit fabric to provide enough room for these modules (Fig. 3e). Based on our design, the minimum distance between each sensor is 1 cm vertically based on the channel width, and 2 cm horizontally based on the interconnect module length. After the whole fabric was drafted, it was then cut for different body parts (Supplementary Fig. 5) using a personalized garment fitting measurement (Supplementary Table 3). Electronic-textile integration was then performed, by threading the electronic strips into the textile channel (Supplementary Fig. 6a), which is further explained in Methods and Supplementary Fig. 7. Finally, the sensor-integrated fabrics were then sewn into a bodysuit to form E-TeCS (Fig. 3f), as illustrated in detail in Supplementary Fig. 6b, with the inside of the E-TeCS shown in Supplementary Fig. 6c, d.

Supplementary Fig. 8 shows the diagram and photographs of electrical connections between the main module and E-TeCS for processing, communication and powering. All of the sensors going horizontally through the stripes are collected with four thin copper wires vertically (Supplementary Fig. 9) through the seams and connected to the main hub (MetaWearR, Mbientlab) through I^2^C protocol. The main hub consists of a microprocessor, BLE module, and rechargeable lithium polymer battery in a compact form. The lithium-polymer battery (401622, HYP) as shown in Supplementary Fig. 8f is rated at 3.7 V, 100 mAh and has a 2 h charging time. The total current consumption, while the main module and all of the sensor nodes are active, is approximately 18.6 mA. With the battery rated at 100 mAh, the working lifetime of our system is around 5 h and 20 min. We can improve this lifetime by using lithium-polymer battery with a higher capacity. As illustrated in Fig. 3g, we sewed conductive snaps that function as a textile-hardware connector to link the I^2^C pins on the microprocessor to the I^2^C wires on the textile. The pluggable mechanism (Supplementary Fig. 8b-e) allows the wireless communication and main processing hardware to be removed during charging of the battery. The I^2^C pins of this micro-controller are wired to the conductive snaps for the textile-hardware interface. Through wireless BLE communication, a computer can access all of the sensor addresses and log their data accordingly. These data can then be stored or visualized in real-time with python Matplotlib and pygame library.

E-TeCS must be personalized to ensure there is sufficient pressure for sensor contact between the textile and skin^42^. Using a disk sensor laminated on the skin, Mahanty and Roemer stated that a pressure of 2 mmHg is sufficient to accurately measure skin temperature, while a larger pressure of up to 20 mmHg will result in an increase of temperature due to the pressure exerted to the local tissue^43^. For wearable comfort, the compression pressure should not be more than 44.1 mmHg, which is close to the average capillary blood pressure of 32.3 mmHg near the skin^44^. As shown in Supplementary Table 3, a set of key tailoring measurements^45^ was used as a reference for the design of the E-TeCS. For pressure measurements, ten circumference points of a subject’s arm and a compression sleeve were measured to calculate the size of the reduction, as calculated in Supplementary Table 4 and illustrated in Fig. 3i. By performing mechanical characterization on the base fabric, we can evaluate the fabric rigidity and model the pressure of elastic fabric around the upper limb region of the human body. These modeled values were also cross-validated with a high-accuracy compression fabric sub-bandage pressure monitor (Kikuhime, TT Meditrade)^46^, as illustrated in Supplementary Fig. 10. Figure 3h presents both the experimental and modeled pressure variations across the sleeve. The pressure values for both cases show a similar trend, with a maximum difference of 3 mmHg when the pressure variations are below 8.5 mmHg. These values, therefore, reflect the compression property of our E-TeCS for on-body sensing with a pressure variations of 2–20 mmHg and ensure a comfortable and reliable contact between the sensors and the skin.

## Discussion

To assess the reliability and electromechanical performance of the serpentine interconnects^47–49^, we performed two types of tests. The first test was a one-time uniaxial stretching of the serpentine interconnect until substrate breakage and conductor rupture. Supplementary Fig. 11a demonstrates the setup for this mechanical test. As shown in Supplementary Fig. 11b-d, the extension of three stretchable interconnects do not influence their resistances (0.32–0.45 Ω) until rupture events at strain values around 79–88%.

**Fig. 4:**
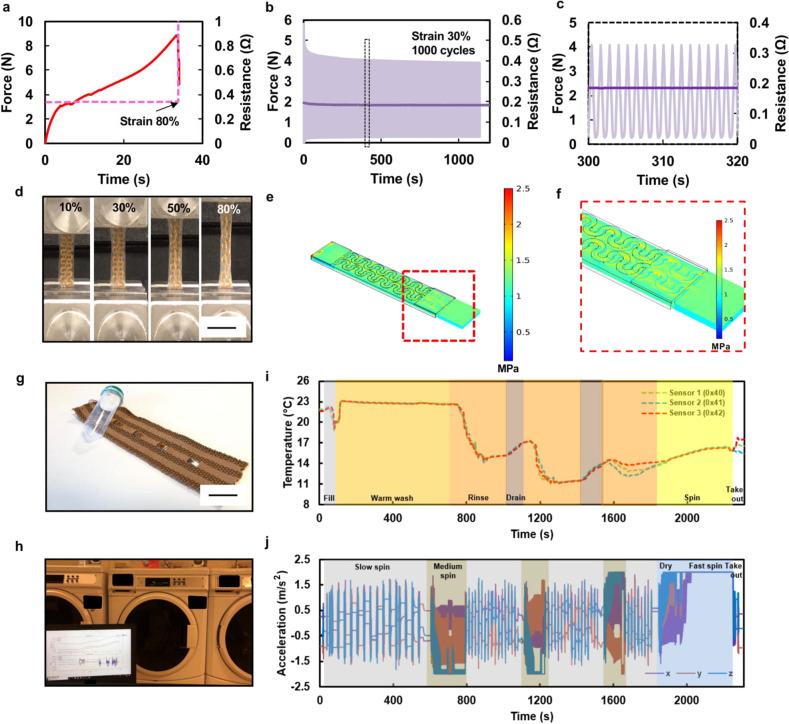
Electrical, mechanical testing, and modeling of interconnects. **a** Instron result of a single uniaxial stretching test until rupture. **b** Time response and its (**c**) magnified view of fatigue cyclic test with a strain of 30%. **d** Image of serpentine interconnects throughout various strain value (scale bar: 2 cm), **e** FEM stress distribution of a serpentine interconnect, and (**f**) Zoomed-in views of stress distribution in (**e**). Real-time washing test. **g** Photograph of the sensorized fabric connected to a BLE system in a sealed, floating chamber (scale bar: 3 cm). **h** Photograph of test setup image. **i** Graph of temperature and (**j**) accelerometer data during the entire washing test.

Two drops can be seen in the load behavior around the rupture points, which occurred due to the sequential breakage of two serpentine lines. A similar response can also be observed in the case of a sensor module connected between two interconnects, with a dimension of 10 mm × 50 mm (Supplementary Fig. 11e–g). All three of the samples’ rupture points localized at around 80% strain, with a stable interconnect resistance of approximately two times that of the case with only one interconnect line (0.6–0.8 Ω). The interconnections do not show any degradation in the electrical property when tested, especially when joined by soldering each connection pad to the sensor module. This test also verifies the robustness of the soldered connections between the interconnects and the sensor modules. Thus, it can be concluded that both types of interconnections are electrically functional and stay highly conductive for a strain value of up to 80%, as shown in Fig. 4d.

The second test we performed was a fatigue test until conductor rupture of both (i) a single interconnect, as well as (ii) a sensor module integrated between two interconnects, which can be used to evaluate the reliability and lifetime of the serpentine interconnects. Most garment distortions happen due to the active movements of the upper body, such as shoulder movements, arm extension, and elbow diameter change^46,50^. According to Hatch, the typical stretchability range of textiles for a tailored garment is 15–25%, for sportswear is 20–35%, and for a form-fit compression garment is between 30–40%^51,52^. Based on these ranges, we expect our E-TeCS to withstand a strain of up to 30%. It was observed that both cases of stretchable interconnects could withstand 1000 stretching cycles at 30% elongation (Supplementary Fig. 12a). Both interconnects show stable, flat low resistance behavior as a conductor throughout the test (Fig. 4b, c and Supplementary Fig. 12e, f). Load versus strain graphs in Supplementary Fig. 12c, d illustrate the viscoelastic–plastic behavior of the TPU^53,54^. As shown in Supplementary Fig. 12b, at the first few cycles, there is a large gap and hysteresis shift of load due to the viscoplastic behavior of TPU, before the mechanical integrity of the TPU weakens and become more elastic at the rest of the stretching cycles. After the fatigue test, both samples showed an elongation of around 10%. Based on this result, there is always be a viscoplastic–elastic adaptation on the first few cycles before the stretchable interconnects achieve a consistent mechanical response. Optimizations in serpentine design, materials choice, and substrate thickness can be performed to improve the durability of this type of stretchable interconnects^48,49^.

The mechanical performance of the serpentine structure was also simulated using commercial FEM package COMSOL Multiphysics 5.0. One end of the serpentine model was applied with a fixed constraint, while a boundary load was applied to the other end of the serpentine model. The top polyimide surface was set to be symmetric^55^. The TPU material is assumed to be hyper-elastic and exhibit viscoelasticity. Stress distribution was simulated for the tensile test with a deformation of 30%. Figure 4e shows the simulated stress distribution across the serpentine sample. The zoom-in views of the deformed samples (Fig. 4f) reveal the maximum normalized stress occurred at arc angle of ±90 °C. The simulation results of 1.8335 MPa has an agreement with experimental tensile strength measurement (1.8755 MPa) at the large deformation region of 30% based on Fig. 4a, with simulation error of less than 10%.

Similar to how we regularly treat our garments, we also designed our electronic textile to be washable for long-term use. Toward this end, we first embedded light-emitting diode (LED, ROHM Semiconductor) strips into the textile channels for a washability test. LED brightness with a supply voltage of 2 V and interconnect resistance were unchanged after the first wash until up to ten wash cycles (Supplementary Fig. 13a, b). The range of resistance values (2.4–2.7 Ω) was as expected, as it was noted on the previous mechanical tests that (Supplementary Fig. 11d) each serpentine has a resistance of 0.32–0.45 Ω and in the fabric sample, and a total of eight serpentine interconnects are connected in series. We observed no flakes or discoloration on the washable encapsulation (PE773, Dupont) after ten washing cycles and liquid chemical treatment (Ultra Stain Release, Tide).

We also conducted a continuous and real-time washing study, where we wove a strip of three temperature sensors and an accelerometer module (Fig. 4g) into a textile patch and put them into an industrial washing machine (MHN33PDCWW0, Maytag Washer), as demonstrated in Supplementary Video 1. Figure 4i, j captures the multimodal sensor data of the entire washing cycle (Fig. 4h) that lasted for 34 min. Since the ‘delicate and knit’ option was chosen, cold water was mostly used during the wash. Throughout the washing test, the textile patch underwent an initial warm wash, three cycles of rinsing, two cycles of draining, and a dry spin at the end. The temperature recordings reflect these events, while the accelerometer readings show four cycles of sequential slow spin, three cycles of continuous medium spin, and a cycle of fast spin for drying mode in the end. It can be observed that towards the end, the accelerometer values are saturated by the medium and fast spin. These tests thus prove the robustness of the encapsulation and interconnections of the system not only mechanically, but also electrically during delicate washing.

The breathability, which is the ability of a fabric to permeate moisture vapor, such as due to sweat or perspiration is one of the most vital comfort factors in garment design^56^. Measurements of daily water vapor transmission in this work follow the standards as described in ASTM E96^57^. Three fabric samples from 100% cotton fabric, 95% polyester and 5% spandex sports fabric, and our own 100% high-flex polyester fabric were cut and sealed to each dish opening using rubber bands (Supplementary Fig. 14a). Accumulated weight loss of each dish was measured daily. From the fitting results, it can be observed that even though our own customized, double-layer knit fabric is thicker (1.9 mm) compared to the cotton (0.4 mm) and sports fabrics (1.1 mm), the breathability of our fabric is still 6.22% higher than the sports fabric, yet 31.04% lower than to the open-air case (Supplementary Fig. 14b and Supplementary Table 5).

**Fig. 5:**
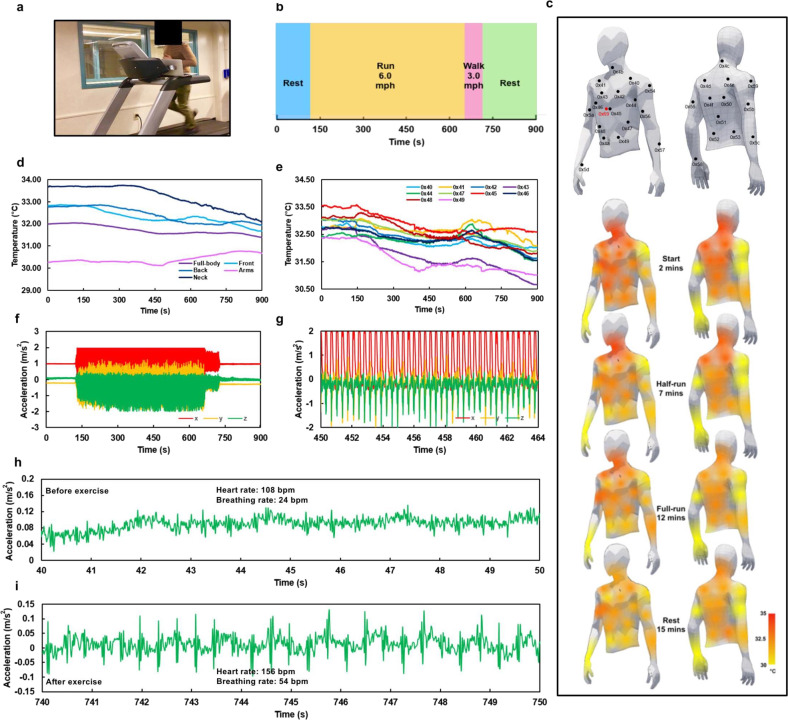
Physical exercise, spatiotemporal physiological mapping, and movement analysis. **a** Photograph of a subject performing the physical exercise task wearing a E-TeCS. **b** Timeline of four separate sections of the physical exercise task. **c** Sensor mapping and body heat-map of the subject throughout the exercise. **d** Full-body and each section of the body skin temperature, and (**e**) anterior skin temperature sensor data during the exercise. All 3-axis accelerometer data (**f**) throughout the entire task. (**g**) in the middle of a graded load test at 6 mph. Raw z-axis sensor reading (**h**) before and (**i**) after the exercise.

Temperature distribution across E-TeCS will enable us to study heat transfer between our skin and environment. Intense physical activity activates the muscle, produces heat in the core element, and initiates vasoconstriction that transfers blood from internal to superficial regions of the body^58^. We can, therefore, monitor temperature change around the body during various dynamic physical activities such as daily activity and exercise, to see how heat dissipation and perspiration influence thermal comfort or athletic performance. We performed an activity test on a subject wearing the E-TeCS (Fig. 5a). A male volunteer with no prior medical history of disease was recruited for participation in this test, and informed, signed consent was obtained from the individual after passing the pre-screening procedure. Figure 5b shows the timeline of the activity tasks throughout the 15 min test.

Supplementary Fig. 15 shows all of the raw temperature sensor data throughout the body during the 15 min running test, while Fig. 5e and Supplementary Fig. 16 provide the calibrated temperature readings according to the linear fit equation found in Supplementary Fig. 3a. Sensor data in these figures are separated in terms of their respective location: on the posterior side, anterior side, both arms, and the neck. In addition, for visualization purposes, Fig. 5c illustrates a body heat-map from the temperature sensor data corresponding to each location. Supplementary Video 2 also demonstrates simultaneous temperature and accelerometer readings while the subject is running. It can be observed that at the start of the activity test, the body heat-map shows a higher temperature profile on the neck, chest, upper-abdomen and upper-back regions, and becomes lower towards the lower-abdomen and lower-back which agrees to a previous study^59^.

In some cases, as illustrated in Fig. 5d, e, we can observe a short increase in temperatures across various body regions before they decrease in trend once the subject started to run at a graded load. A sudden change in exercise intensity increases cutaneous blood flow and releases heat, resulting in an increase of core and skin body temperature. This phenomenon occurs until perspiration starts and sweat evaporates from the eccrine glands of the skin, providing a cooling effect and decreasing the skin temperature throughout^60^. As the sweat permeates through the fabric, the temperature tends to stabilize towards the end of the resting period. We can see that temperature around the posterior, especially at the arms, does not show a significant trend, which may be due to the local heat flux and blood flow that mostly originate from primary organs around the central region^59^.

To confirm our exercise results, we conducted a second running task and performed IR thermography (Duo R, FLIR) on the same subject without the E-TeCS. Supplementary Fig. 17 shows the body heat-map at the anterior, posterior, and lateral view from the thermal camera throughout the running test at a graded load. The color change indicates a reduction in temperature across the whole body caused by the sweat, with an incremental increase while resting from minute 12 to 15. Even though the thermal camera has a higher resolution (160 pixels x 120 pixels) compared to the E-TeCS (30 points), it has a relatively low thermal accuracy (±5 °C) compared to our body temperature sensor with accuracy and precision of approximately 0.1 °C and 0.01 °C, respectively^61^. Thermal images from the commercial IR thermography camera show a body temperature spread of 29.8 to 30.85 °C, while wearing the E-TeCS results in a temperature spread of 28.5–34.7 °C. The latter range is closer to typical body temperature range during normal activity and intense physical exercise^62^.

Accelerometer data and mechano-acoustic waveforms from the activity test are also presented in Fig. 5f–i. Figure 5f shows all 3-axis accelerometer data for the entire 15 min of the task. We can observe the intensity of the task, shown as periodic 3-axis waveforms that can be counted to 174 steps per minute, representing running at 6 mph (Fig. 5g). The increasing acceleration when the subject started running at a graded load (Supplementary Fig. 18a) and the transitioning acceleration as the subject slowed down to walking at three mph, corresponding to 120 steps per minute (Supplementary Fig. 18b), are also clearly visible. By zooming into the *z*-axis acceleration at rest, we can observe the mechano-acoustic waveforms, triggered by the subtle contraction and relaxation of the heart, lung, and diaphragm (Fig. 5h, i). Both figures represent raw data before any further processing, such as filtering. Before the exercise, we can see a clear breathing waveform in Fig. 5h, corresponding to 4 breaths per 10 s (24 breaths per minute) with small peaks of the beating heart of 18 spikes per 10 s (108 heartbeats per minute). After the subject performed a graded load exercise, a large amplitude of mechano-acoustic vibrations from the heart was visible due to the increase in cardiac output. As physical exercise intensity increases, the heart needs to pump more blood and oxygen supply to meet the demand of the body’s muscles. The lung and respiratory system also respond to the intensity, with an increased breathing rate to compensate for the oxygen requirement of the body to release energy^63,64^. In correlation to the activity of these organs, after the exercise, both heart rate and breathing rate increased to 156 bpm and 54 bpm, respectively (Fig. 5i).

In summary, we have merged flexible-stretchable electronics with customized knit fabrics to develop an E-TeCS for distributed on-body sensing in vivo. Large-scale manufacturing of flexible printed circuit boards and knit fabrics and modular sensor networks enable a high-throughput, scalable system, resulting in: (1) large-area sensor coverage, and (2) a versatile platform for multimodal sensor integration. Not only did we produce our own fabric structures and patterns, but with garment design and patterning techniques, we also tailored the fabric into a suit for a tight fit, yet comfortable for conformal attachment to the curvature of the body. The engineered compression pressure across the body ensures each sensor’s contact to the skin and minimizes dislocation from the sensing points. As our final prototype, we integrated 31 sensor islands into the tailored E-TeCS, including 30 temperature sensors spread across the upper-body region, and one accelerometer placed right below the sternum. Intense physical exercise was conducted to demonstrate the ability of E-TeCS to perform continuous spatiotemporal temperature sensing, as well as simultaneous mechano-acoustic sensing for the estimation of heart rate and breathing rate. Compared to IR thermography used in this work, our approach enables high-accuracy skin temperature sensing without being spatially limited by the camera’s view or the need to be naked, expanding its applications in wearable sensing “on-the-go”. The accelerometer could also detect subtle heart rate, respiration, and body movements for physical activity and physiological monitoring. Future studies may focus on incorporating additional sensing modalities such as humidity, pressure, optical, ultrasonic, gas, magnetic field sensors, and so on, demonstrating the E-TeCS capability during various activities outside the lab, and performing further optimization for electromechanical and washability study. The collective design and integration approach of E-TeCS, as well as the underlining experimental and implementation studies would be of interest in the development of flexible-stretchable and textile electronic systems. The multi-modal, multi-functional framework of E-TeCS will enable a new strategy of personalized telemedicine for rapid prototyping and deployment, especially during extreme conditions such as a pandemic or natural disaster relief efforts. It could advance mobile, comfortable, and continuous physiological and physical activity monitoring, with potential implications in healthcare, rehabilitation, and sports science not only in the hospital and laboratory, but also in home-care settings and eventually in outer-space applications.

## Methods

### Fabrication of the sensing island

The structure of a sensor module in Fig. 1e consists of two-layer flexible PCB (FPCB, KingCredie) with 18 μm thick Cu traces, 28 μm thick base polyimide (PI) substrate, and 28 μm thick PI outer shell. The MAX3025 (Maxim Integrated) sensor IC, 850 μm in thickness is soldered into the pads with 75 μm thick PI stiffener as a support structure and encapsulated with 150 μm thick washable encapsulant (PE773, Dupont). The entire module is then encapsulated in a TPU shell (TE-11C, Dupont) with 100 μm thickness for each top and bottom layer. For cross-sectional imaging, an electronic device woven into a fabric channel is submerged and cured in a Polydimethylsiloxane mix (Sylgard 184, Sigma-Aldrich) with base and curing agent ratio of 1:10 bath. We then cut the molded device with a circular cold saw (CS-350, Kalamazoo) at the middle of the chip. Finally, we polished the device using the side of a rotating circular blade (Wilton Corporation).

### Development of the customized, digital knit fabrics

The knit fabrics were developed by a digital flat two-bed knitting machine (Super-J 212, Matsuya). Two yarn carriers were used in order to make two layers of weft-knit fabric (Fig. 5e). Weft knitting is a method of forming a fabric in which the loops are made in a horizontal way from a single yarn. With a two-bed knitting machine, single layer fabric can be realized by interlocking. Interlocking uses two sets of needles that knit back-to-back in an alternate sequence to create two sides of the fabric that are exactly in line with each other, forming one layer. Each yarn carrier holds 2-ply (75 denier each ply) of high-flex polyester yarns. Textile channels for electronic integration were knitted by allowing both the front and back needle beds to knit simultaneously and by making a spacer fabric with a hollow channel. The number of wale lines, which is 20 in this spacer fabric defines the width of the opening of around 1 cm while the course line number defines the width of the entire knit fabric (Fig. 5c). The rest of the fabric was formed through interlocking. Solder-tip melting (WP80, Weller) was performed to open the channels for the exposed part of the sensor modules with a distance of 1.5 cm.

### Fabrication of the E-TeCS

After the pattern was drafted, the knitted fabric was laser-cut (Helix 75W, Epilog) with the open channels positioned in a horizontal orientation (Supplementary Fig. 5). The horizontal measurements (e.g. neck circumference, waist circumference, thigh circumference) were reduced by around 10% depending on the dimension to ensure a tight fit. The optimal amount of strain can be determined after further testing the yield strain of the stretchable interconnects, the compression pressure, and on the fit of the suit. A seam allowance of 1.5 cm was used on the pattern pieces. The shirt (Fig. 3f) consists of a front, a back, two sleeves, and polo neckpieces. The raw edges of the seams were joined together using a zig-zag stitch with a sewing machine (CG590, Singer) as an overlocking stitch (Supplementary Fig. 6b).

### Integration of electronic textile

As illustrated in Supplementary Fig. 7, after the sensor-interconnects modules bonding by hot-melt soldering (Pb-free #4900–112 G, MG Chemicals), the sensor electronics were encapsulated (PE773, Dupont) by using medical and semiconductor grade epoxy resin that is machine-washable for both mechanical and electrical protection. The electronic strips were then further encapsulated in a stretchable outer shell, in which two films of thermoplastic polyurethane (TPU TE11-C, Dupont) are laminated and each side of the TPU is bonded with heat (150 °C). After that, the stretchable electronic strips can be integrated into one of the textile channels through manual weaving (Supplementary Fig. 6a). Every sensor is exposed through the opening and glued to the textile with washable fabric glue (OK to Wash-It, Aleene). Four power and signal wires from the main hub were threaded to every end of these strips to connect the microprocessor to all available sensors (Supplementary Fig. 9).

### Fabric rigidity test

Four digitally knit fabric patches were cut in 5 cm × 10 cm and used as samples for tensile strength test using a commercial mechanical tester machine (Instron 5943). The samples were extended with a speed of 200 mm/min using a 0.5 kN load cell. Load and extension data were recorded until the samples ruptured. We consider a typical stretch range for compression garments, which is the first portion of a load–extension curve (5–35%) to calculate the rigidity of our fabric^42^.

### Compression pressure modeling

To model the pressure in a compressive garment, we first define the rigidity of the elastic fabric material as

1 $${\rm{El}} = \frac{{\Delta T}}{{\Delta {\rm{St}}}},$$

where *T* is the fabric tension per unit length in gf/cm and St is the fabric extension. Assuming that we have a tubular fabric covering a cylindrical tube, the fabric extension and the size of the reduction (Re) are given by

2 $${\rm{St}} = \frac{{R - r}}{r},$$

3 $${\rm{Re}} = \frac{{R - r}}{R},$$

4 $${\rm{St}} = \frac{{{\rm{Re}}}}{{1 - {\rm{Re}}}},$$

where *R* is the radius of a cylindrical tube and *r* is the radius of tubular elastic fabric (*R* > *r*). By applying Laplace’s law, the pressure (*P*) in gf/cm^2^ can be defined as

5 $$P = \frac{T}{R}.$$

Expressing *C* as the circumference of the cylindrical tube gives us

6 $$T = \frac{C}{{2\pi }}P.$$

Substituting parameters in Eqs. (1) and (4) with Eq. (5) results in

7 $$\frac{{{\rm{Re}}}}{{1 - {\rm{Re}}}}{\rm{El}} = \frac{C}{{2\pi }}P,$$

8 $${\rm{Re}} = \frac{1}{{1 + \frac{{2\pi {\rm{El}}}}{{C_{{\rm{tube}}}P}}}}.$$

Since the human body model is not a perfectly cylindrical tube, we define a compression factor to define relationship between the circumference of the human body and cylindrical tube

9 $$C_{{\rm{tube}}} = \frac{{C_{{\rm{body}}}}}{{{\rm{CF}}}}.$$

Rearranging Eq. (9) into Eq. (8) gives us the final pressure value of elastic fabric for compressive garment purposes

10 $$P = \frac{{2\pi {\rm{El}}\left( {{\rm{CF}}} \right)}}{{C_{{\rm{body}}}}}\frac{{{\rm{Re}}}}{{1 - {\rm{Re}}}}.$$

In order to find the compression pressure throughout the body, we initially need to study the tensile properties and calculate the rigidity of our fabric material. We consider a typical stretch range for compression garments, which is the first portion of a load–extension curve (5–35%), to calculate the rigidity of our fabric. By using Eq. (1), the rigidity of each fabric is calculated to be 662.8, 846.4, 773.6, and 716.84 gf, respectively (Supplementary Fig. 10a). A study on 34 human subjects revealed that compression factor (CF) for the upper limb of a human body is 0.9^65^. Using the aforementioned values in Eq. (10), we can then estimate the pressure of elastic fabric around the upper limb region of the human body.

### Electromechanical testing of interconnects

To assess the reliability and electromechanical performance of the serpentine interconnects, we performed two types of tests. The first test is one-time uniaxial stretching until substrate breakage and conductor rupture. Supplementary Fig. 11a demonstrates the setup for this test. A commercial mechanical tester (Instron 5943) with a 0.5 kN load cell was used. Load and extension data were recorded using a crosshead speed of 1 mm/s until 100% extension of the original length of the samples. The prepared samples were the interconnect modules with two serpentine lines, and dimensions of 10 mm × 20 mm. Resistance was measured with an LCR meter (E4980A, National Instrument) connected to the integrated sensor leads with probes. Via a common I/O interface (BNC-2111, National Instruments), the load, extension, and resistance data were synchronously obtained and logged.

### Wireless communication

All of the sensors going horizontally through the stripes were collected by four thin copper wires, which aligns and inserts vertically through the seams and is connected to the main hub (MetaWearR, Mbientlab) via I^2^C protocol. We sewed conductive snaps that function as a textile-hardware connector to link the I^2^C pins on the micro-controller to the I^2^C wires on the textile. The pluggable mechanism allows the hardware to be removed during charging of the battery. Through wireless Bluetooth communication, a computer can access all of the sensor addresses and log their data accordingly. These data can then be stored or visualized in real-time with python Matplotlib and pygame library.

### Temperature sensor characterization

A temperature sensor was embedded in a piece of fabric and encapsulated by a thermally conductive epoxy (PE-773, Dupont) and thermoplastic polyurethane (TE-11C, Dupont). After being embedded into the fabric, the electrically packaged sensor was placed on the surface of a hot plate with direct contact. The sensor was heated from 25 °C to 50 °C on the hot plate at a ramp rate of 300 °C/h. While the temperature on the anodized Aluminum plate ramped up and was being recorded (25 Hz) by a high-accuracy IR camera (PI 400i, Optris) with thermal sensitivity of 40 mK and an accuracy of ±2%, a micro-controller (Arduino UNO) simultaneously gathered data from all three flexible temperature sensors (33 Hz) and logged these sensor data to a computer. The IR temperature data from a point near the flexible temperature sensors on an anodized Al plate were then compared to the sensor temperature data (*n* = 4) at every 1 °C elevation of temperature.

### Inertial sensor characterization

We assembled, encapsulated, and integrated an accelerometer module within a customized fabric (Fig. 2j). The simultaneous seismocardiography and electrocardiography test was performed while the subject was laying on a bed in a relaxed state. We sampled the accelerometer-embedded fabric’s *z*-axis data (100 Hz), which was wired to an Arduino UNO through I^2^C communication, alongside a commercial ECG (1000 Hz) and respiration (25 Hz) strap (Zephyr BioPatch, Medtronic) as a cross-validation device.

### Washability testing

The electronic textile patch was connected to a BLE module (MetaWearR, Mbientlab) that is sealed inside a tube with clear silicone glue (RTV Silicone, Dynatex). This setup then went through a full washing cycle. As shown in Supplementary Video 1, the “delicate and knit” option was chosen, and logging and real-time streaming of sensor data from inside the industrial washing machine (MHN33PDCWW0, Maytag Washer) during the complete cycle was performed. Throughout the washing with 20 g of standard detergent (Ultra Stain Release, Tide), the textile patch underwent an initial warm wash, three cycles of rinsing, two cycles of draining, and a dry spin at the end. After that, the electronic patch was dried for an hour by exposing it to warm airflow generated by 1500 W ceramic portable heater (CD09250, Lasko) in the high setting.

### Breathability testing

Measurements of daily water vapor transmission in this work follow the standards as described in ASTM E96^57^. Four 70 mm diameter by 50 mm height glass Petri dishes were prepared, each filled with 40 g of water. Three fabric samples from 100% cotton fabric, 95% polyester and 5% spandex sports fabric, and our own 100% high-flex polyester fabric were cut and sealed to each dish opening with rubber bands (Supplementary Fig. 14a). Accumulated weight loss of each dish was measured daily for eight days at room temperature (21 °C) and 50% humidity, using a precision analytical scale (ME54TE, Mettler Toledo). This weight loss (Δ*W*) is the amount of water vapor that has transmitted through the fabrics and evaporated. The water vapor transmission rates (WVTR) can be calculated as follows:

11 $${\rm{WVTR}} = \frac{{\Delta W({\rm{g}}/24\,{\rm{h}})}}{{A({\rm{m}}^2)}},$$

where Δ*W* is the slope of the weight change in grams (g) every after 24 h and *A* is the transmission surface area in m^2^.

### Activity study design

We performed an activity test on a subject wearing the tailored E-TeCS (Fig. 5a). All experiments were conducted in compliance with the guidelines of IRB and were reviewed and approved by the Massachusetts Institute of Technology Committee on the Use of Humans as Experimental Subject (COUHES Protocol 1901656745). A male volunteer with no prior medical history of chronic cardiovascular, skin, mental health disease, or physical disability was recruited for participation in this test, and informed, signed consent including consent of photography during the test was obtained from the individual after passing the pre-screening procedure. The subject was asked to stand still on a treadmill for 2 min before commencing the physical exercise test (Supplementary Video 2). The subject then started to run at a graded load of 6 mph for 9 min, before slowing down to 3 mph for 1 min. Finally, the subject stopped the treadmill and rested by standing for 3 min until the test ended. During the entire test, the E-TeCS accessed, captured, and sent multi-nodal body temperature (1 Hz) and IMU (100 Hz, accelerometer *x*, *y*, and *z*-axis) data to a computer through BLE communication for logging. The subject performed the same test for a second time and was naked, without wearing the E-TeCS for validation with an IR camera (Duo R, FLIR).

### ECG and respiration filter

A finite impulse response low-pass filter with *F*_s_ of 1000 Hz, *F*_pass_ of 60 Hz, *F*_stop_ frequency of 180 Hz, *D*_pass_ of 0.05, and *D*_*s*top_ of 0.0001, where *D* is the deviation (ripple) vector, is used to process the raw data by eliminating low-frequency respiratory waveforms. For respiratory waveform, FIR low-pass filter with *F*_s_ of 1000 Hz, *F*_pass_ of 1 Hz, *F*_stop_ frequency of 2 Hz, *D*_pass_ of 0.0005, and *D*_stop_ of 0.000001 are used instead for eliminating high-frequency signals due to heart-beat events and getting the DC component of the signals.

## Supplementary information

Supplementary Information

Video 1

Video 2

## Data Availability

The data that support the findings of this study are available from the authors on reasonable request. The authors declare that the data supporting the findings of this study are available within the article and the corresponding Supplementary Information File.

## Code availability

The custom code and mathematical algorithm that support the findings of this study are available from the authors on reasonable request. The authors declare that the data supporting the findings of this study are available within the article and the corresponding Supplementary Information File.
