# Supplementary material for: A tailored, electronic textile conformable suit for large-scale spatiotemporal physiological sensing in vivo
Source: Npj Flex Electron. 2020 Apr 23;4(1):5. doi: 10.1038/s41528-020-0068-y (PMC7222957; doi:10.1038/s41528-020-0068-y)
Supplement: Supplementary file 1 — Supplementary Information [file 41528_2020_68_MOESM1_ESM.pdf]

A tailored, electronic textile conformable suit for large-scale spatiotemporal physiological sensing *in vivo*

Irmandy Wicaksono[1], Carson I. Tucker[2], Tao Sun[1], Cesar A. Guerrero[3], Clare Liu[3,4], Wesley M. Woo[3], Eric J. Pence[3], & Canan Dagdeviren[1*]

[1]*Media Lab, Massachusetts Institute of Technology, Cambridge, 02139, MA, USA*
[*]Email: canand@media.mit.edu

[2]*Mechanical Engineering, Massachusetts Institute of Technology, Cambridge, 02139, MA, USA*

[3]*Electrical Engineering and Computer Science, Massachusetts Institute of Technology, Cambridge, 02142, MA, USA*

[4]*Architecture and Planning, Massachusetts Institute of Technology, Cambridge, 02139, MA, USA*

Approach	Sensing Modality	Sensing Area and Number	Naked/Clothing	References
**IR Camera**	Temperature	Full-body, High-resolution	Naked	Tanda [1]
**NFC sticker sensors**	Temperature and Pressure	Full-body, 65 sensors	Naked/Clothing	Han *et al*. [2]
**Semi-disposable patch**	Respiration and Electrocardiography	Upper-body, 3 sensors	Naked/Clothing	Pantelopoulos *et al*. [3]
**Woven sensors**	Temperature, Electrocardiography, and Voice monitoring	Upper-body, 4 sensors	Clothing	Park and Jayaraman [4]
**E-TeCS**	Temperature, Heart-rate, and Respiration	Upper-body, 31 sensors	Clothing	This work

Supplementary Table 1: List of distributed and/or multi-modal sensing demonstrated in the literature, with their comparison to E-TeCS.

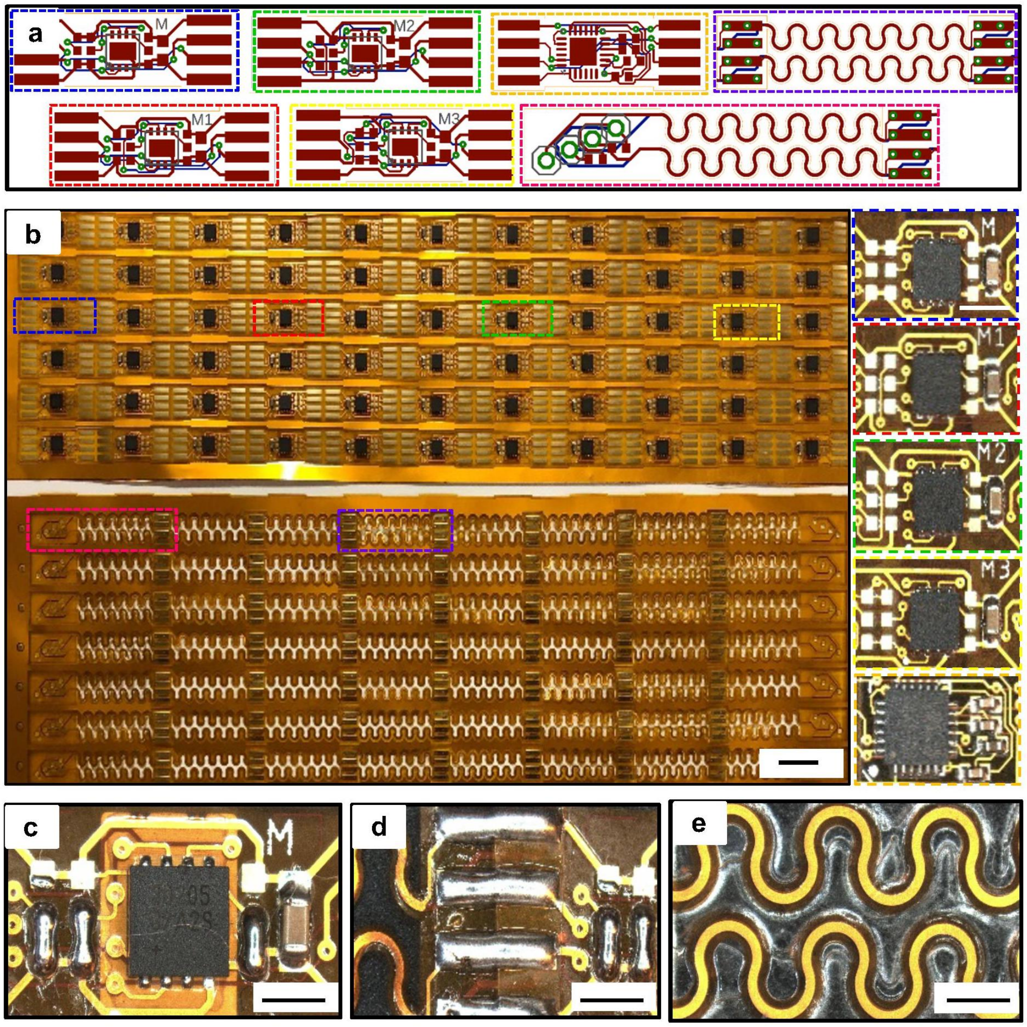

Supplementary Figure 1: Mass-manufacturing of flexible-stretchable printed circuit boards. a) The design of flexible PCB modular sensor and interconnects layout. Photograph of b) temperature sensors, accelerometer, and interconnects assortment including their various layouts for sensor addressing (right, scale bar: 1 cm), c) jumper connection to define a sensor address on MAX30205 (scale bar: 2 mm), d) sensor-interconnects solder connections (scale bar: 2 mm). e) Photograph of interconnects embedded in a TPU film (scale bar: 2mm).

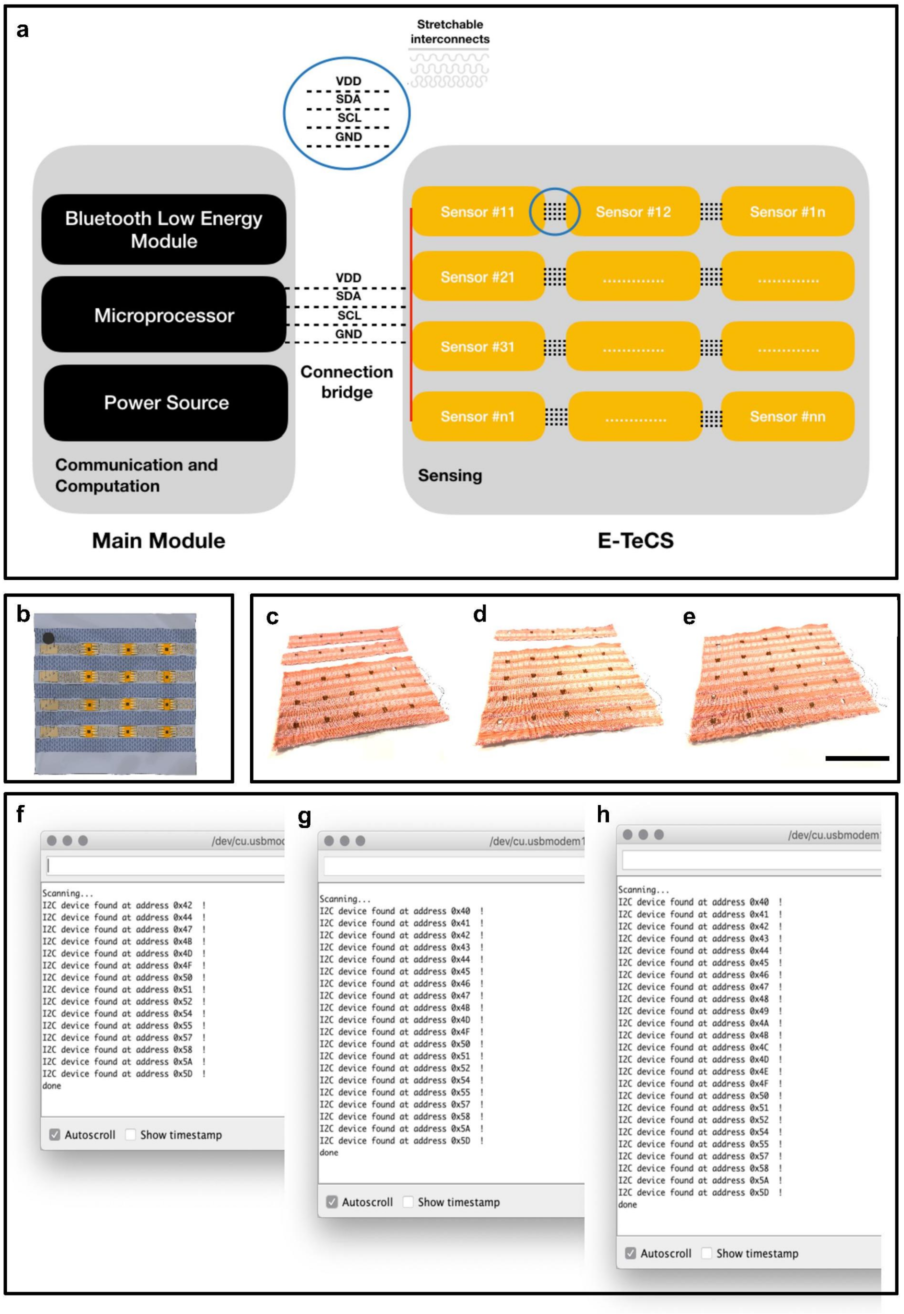

Supplementary Figure 2: Scalable electronic textile system. a) Modular sensor network architecture. b) Illustration of the sensor networks connection on fabric. c-e) Demonstration of scalability of the electronic textile (scale bar: 5 cm) with f-h) more sensors detected in the computer as more electronic textile elements are joined.

A2	A1	A0	Slave address (hex)
GND	GND	GND	90h
GND	GND	VDD	92h
GND	GND	SCL	82h
GND	GND	SDA	80h
GND	VDD	GND	94h
GND	VDD	VDD	96h
GND	VDD	SCL	86h
GND	VDD	SDA	84h
GND	SCL	GND	B4h
GND	SCL	VDD	B6h
GND	SCL	SCL	A6h
GND	SCL	SDA	A4h
GND	SDA	GND	B0h
GND	SDA	VDD	B2h
GND	SDA	SCL	A2h
GND	SDA	SDA	A0h
VDD	GND	GND	98h
VDD	GND	VDD	9Ah
VDD	GND	SCL	8Ah
VDD	GND	SDA	88h
VDD	VDD	GND	9Ch
VDD	VDD	VDD	9Eh
VDD	VDD	SCL	8Eh
VDD	VDD	SDA	8Ch
VDD	SCL	GND	BCh
VDD	SCL	VDD	BEh
VDD	SCL	SCL	ACh
VDD	SCL	SDA	Ach
VDD	SDA	GND	B8h
VDD	SDA	VDD	BAh
VDD	SDA	SCL	AAh
VDD	SDA	SDA	A8h

Supplementary Table 2: MAX30205 temperature sensor I^{2}C addressing

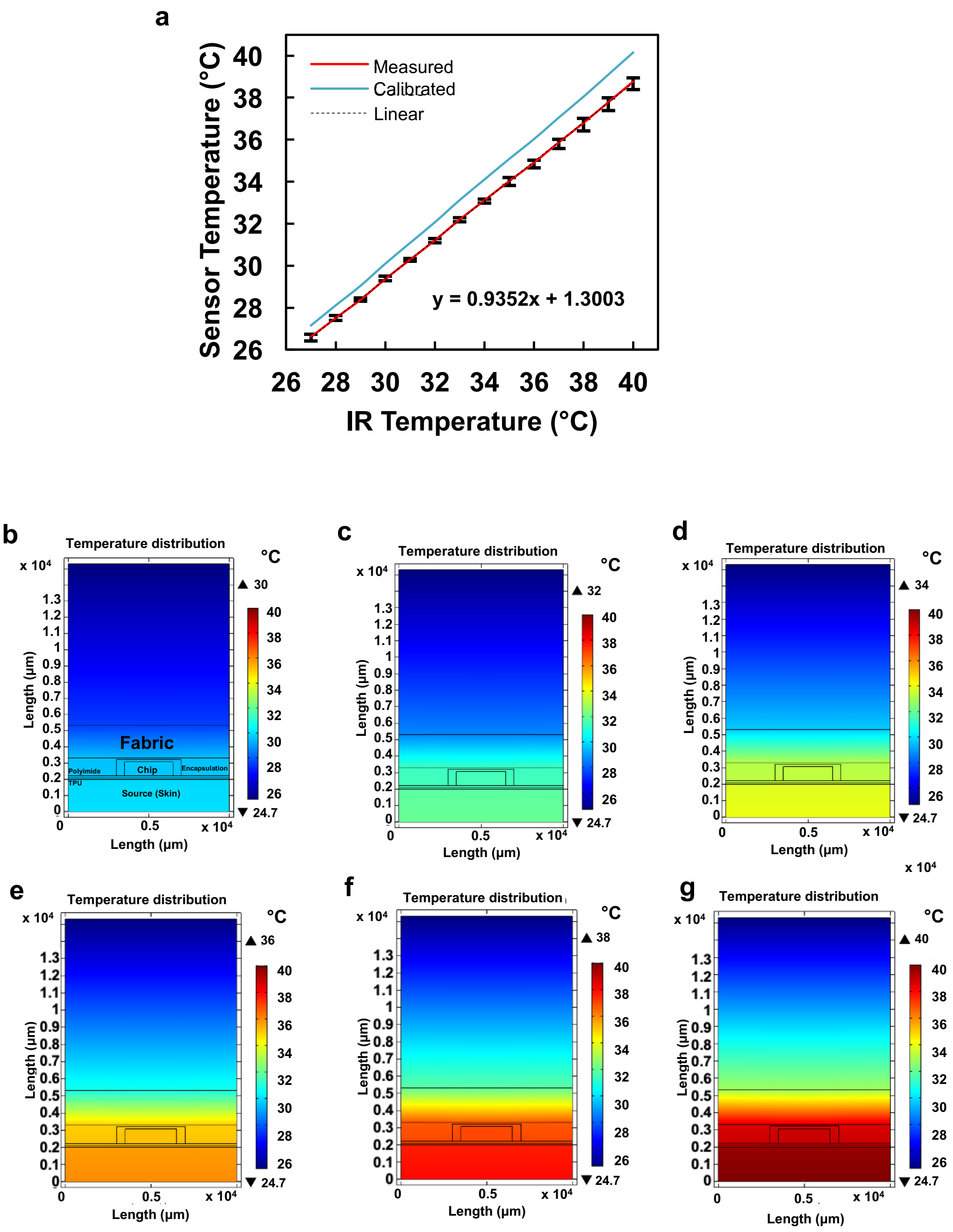

Supplementary Figure 3: Simulation results. a) Linear fitting graph of temperature characterization using IR camera with the calibrated result. b-g) FEM thermal distribution results with a heat source (skin) ranging from 30 to 40 °C to find the effective temperature right below the chip.

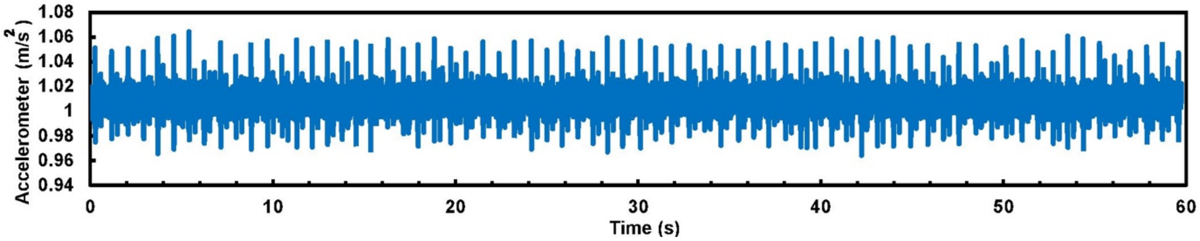

Supplementary Figure 4: Raw accelerometer z-axis data before the heart-beat and respiratory waveform filtering.

Dimension	Measurement (cm)	Desired strain (%)	Pattern Dimension (cm)
**front length**	51.00	0	51.00
**back length**	52.00	0	52.00
**neck circumference**	37.00	10	33.64
**armhole depth**	20.00	0	20.00
**armhole circumference**	49.00	10	44.55
**elbow circumference**	27.50	10	25.00
**wrist circumference**	17.00	10	15.45
**sleeve inseam**	38.00	0	38.00
**shoulder to bicep**	16.50	0	16.50
**bicep to elbow**	10.00	0	10.00
**elbow to wrist**	26.50	0	26.50
**shoulder to wrist length**	56.00	0	56.00
**shoulder width back**	45.00	10	40.91
**center back**	39.00	0	39.00
**bust**	88.00	10	80.00
**upper waist circumference**	81.00	10	73.64
**waist**	76.00	10	69.09
**hip circumference**	84.00	10	76.36
**neck to bust**	18.50	0	18.50
**neck to upper waist**	29.50	0	29.50
**neck to waist**	38.00	0	38.00
**neck to upper hip**	47.00	0	47.00
**neck to hip**	54.00	0	54.00

Supplementary Table 3: Measurement of the body and the calculated pattern dimension for the development of compression garment.

Location	Distance from upper shoulder (cm)	Sleeve circumference (cm)	Arm circumference (cm)	Strain (%)	Reduction (%)
**1**	47	20.0	20.5	2.50	2.44
**2**	43	21.2	24.3	14.62	12.76
**3**	39	23.0	26.9	16.95	14.50
**4**	35	24.4	27.4	12.29	10.95
**5**	31	25.8	26.5	2.71	2.64
**6**	27	27.0	27.5	1.85	1.82
**7**	27	27.0	29.0	7.40	6.89
**8**	23	28.0	30.0	7.14	6.67
**9**	19	29.0	31.0	6.89	6.45
**10**	15	29.5	31.5	6.78	6.35

Supplementary Table 4: Measurements of the subject's arm and the fabric sleeve circumference for strain and reduction calculation.

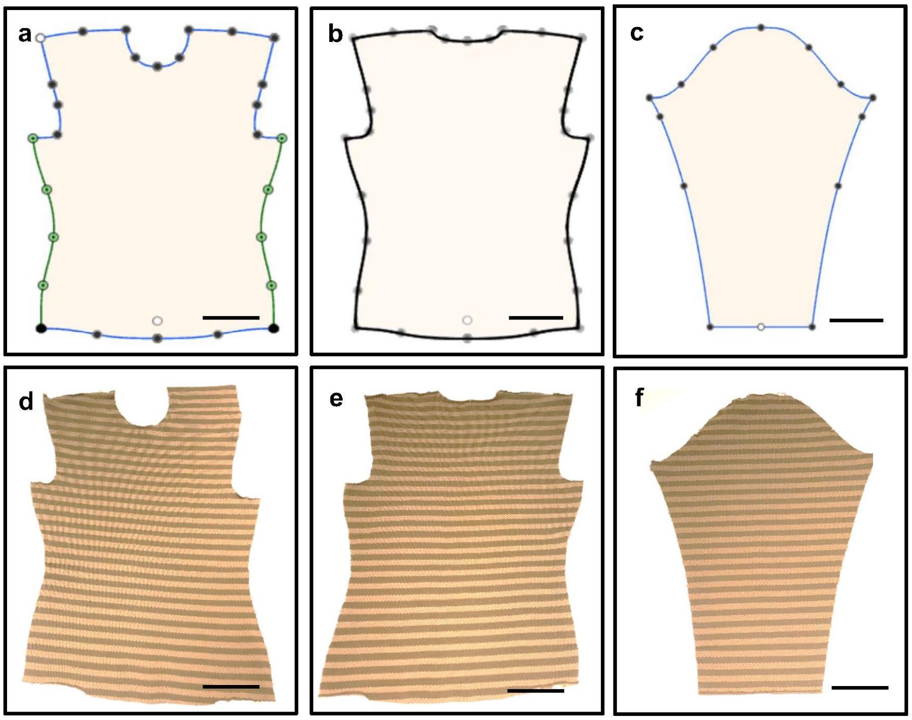

Supplementary Figure 5: AutoCAD drawing of the a) front, b) back, c) sleeve piece and the resulting d) front, b) back, c) sleeve piece after cutting from the customized fabrics (scale bar: 10 cm).

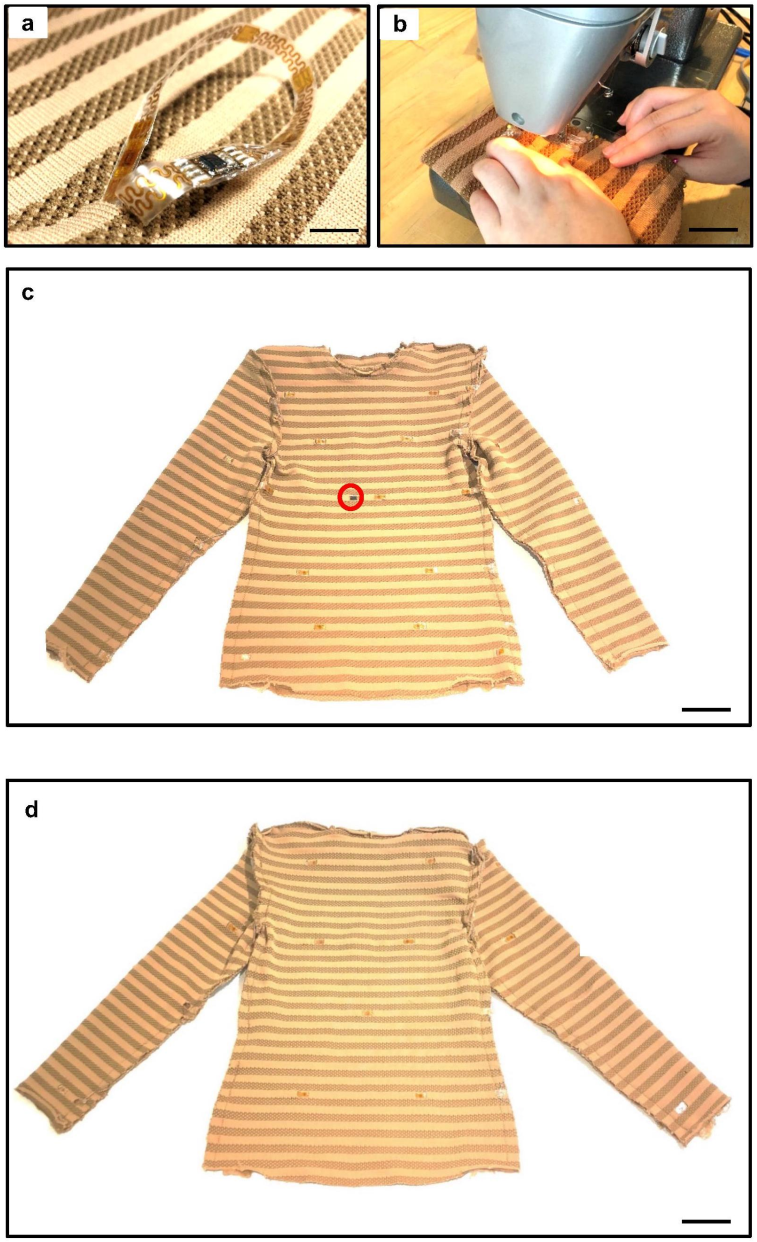

Supplementary Figure 6: Integrating electronic strips and sewing the seams. a) Photograph of a stretchable sensor strip being woven (scale bar: 1 cm). b) The sewing process of the sleeve piece (scale bar: 3 cm). **The inside view of a tailored E-TeCS.** c) front-side, and d) back-side showing the integration of accelerometer (circled in red) and multiple temperature sensors (scale bar: 5 cm).

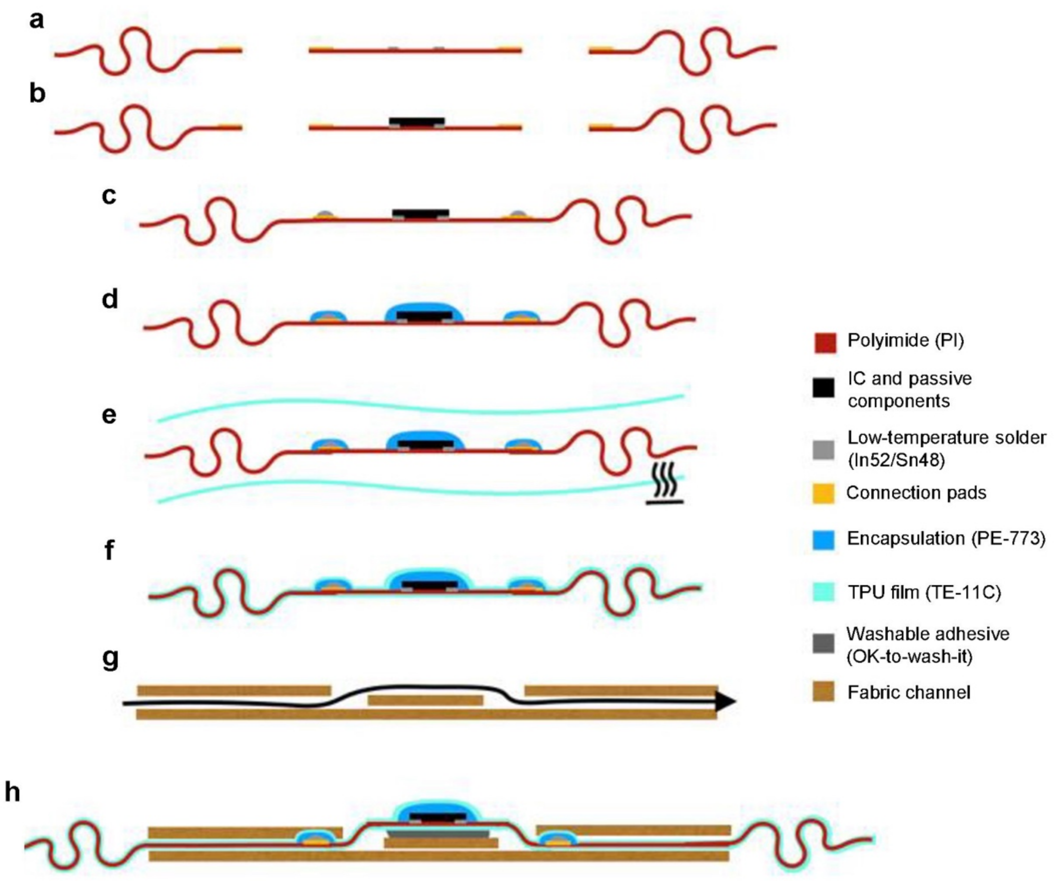

Supplementary Figure 7: Illustration of electronic textile integration process flow. a) Unpopulated sensor and interconnect modules. b) Populating the sensor chips to the islands. c) Interconnects bridge and sensors island are bonded together through soldering. d) Encapsulation of ICs and soldered connections. e) Lamination of TPU on both sides and only both edges with hot air gun. f) After TPU lamination. g) Weaving of electronic strips into textile channels (Black arrow shows the direction of weaving). h) Sensor island exposed through the opening for skin contact with adhesion by fabric glue at the bottom.

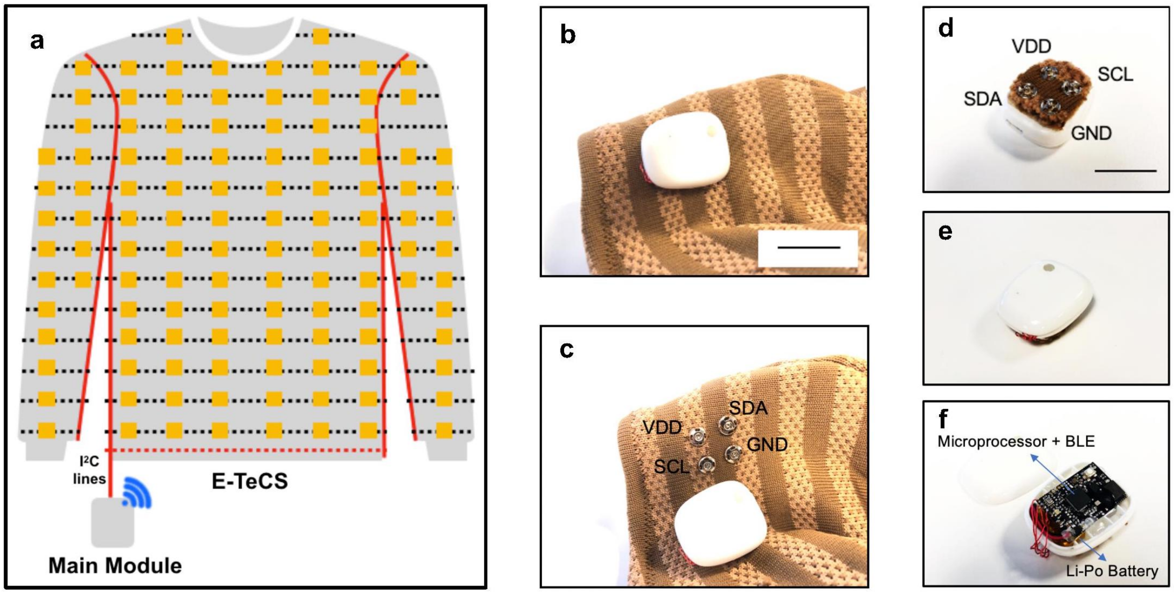

Supplementary Figure 8: E-TeCS connectorization. a) Concept diagram of system-on-textile sensor network connected to a main module (MetaWearR, MbientLab), b) main module connected to the E-TeCS prototype (scale bar: 3 cm), c) main module disconnected to the E-TeCS showing female connectors for I²C powering and communication d) The back of the main module showing I²C male connectors (scale bar: 3 cm), e) The main module enclosed in a packaging, f) Inside the main module showing microprocessor, BLE module, and lithium polymer battery.

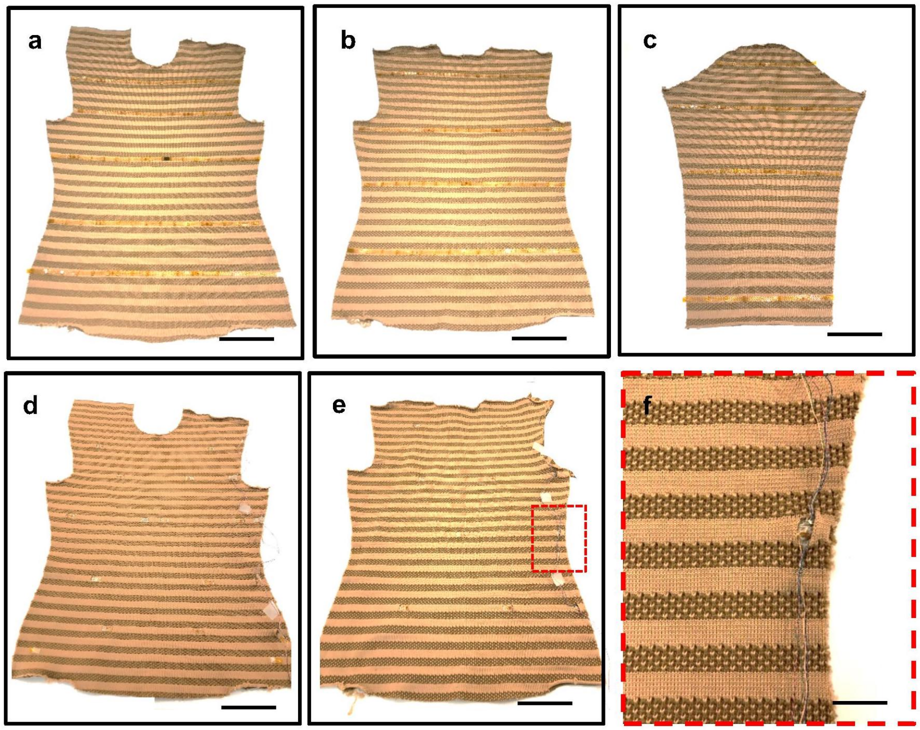

Supplementary Figure 9: Photographs of electronic-integrated fabric piece. a-c) The front, back, and sleeve piece with every flexible-stretchable electronic strip on their position before and d-e) after weaving (scale bar: 10 cm). f) Four thin copper wires hidden at the seam to connect all of the strips to the main hub for processing and communication (scale bar: 1 cm).

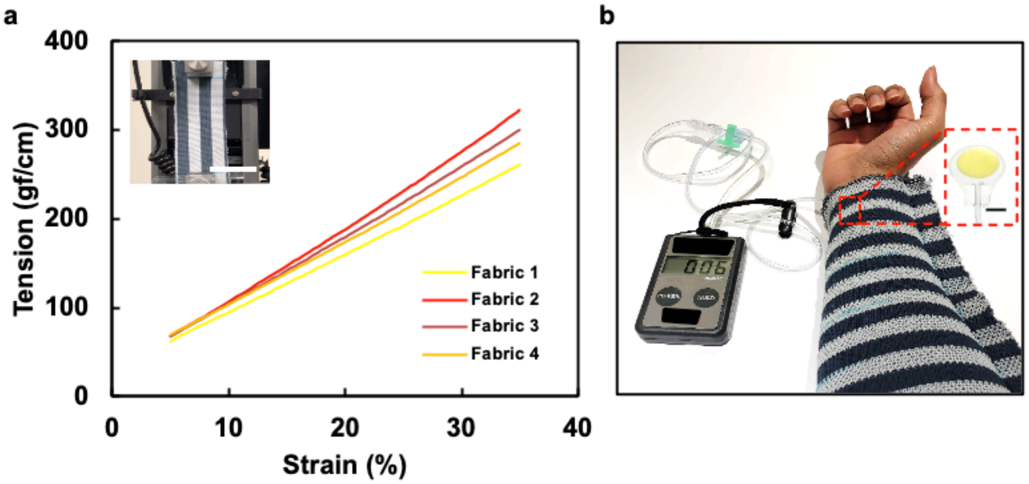

Supplementary Figure 10: Compression pressure profile measurement. a) The graph of fabric rigidity measurement through Instron mechanical test. b) Photograph of sub-bandage pressure monitoring of an arm sleeve (inset, scale bar: 2 cm).

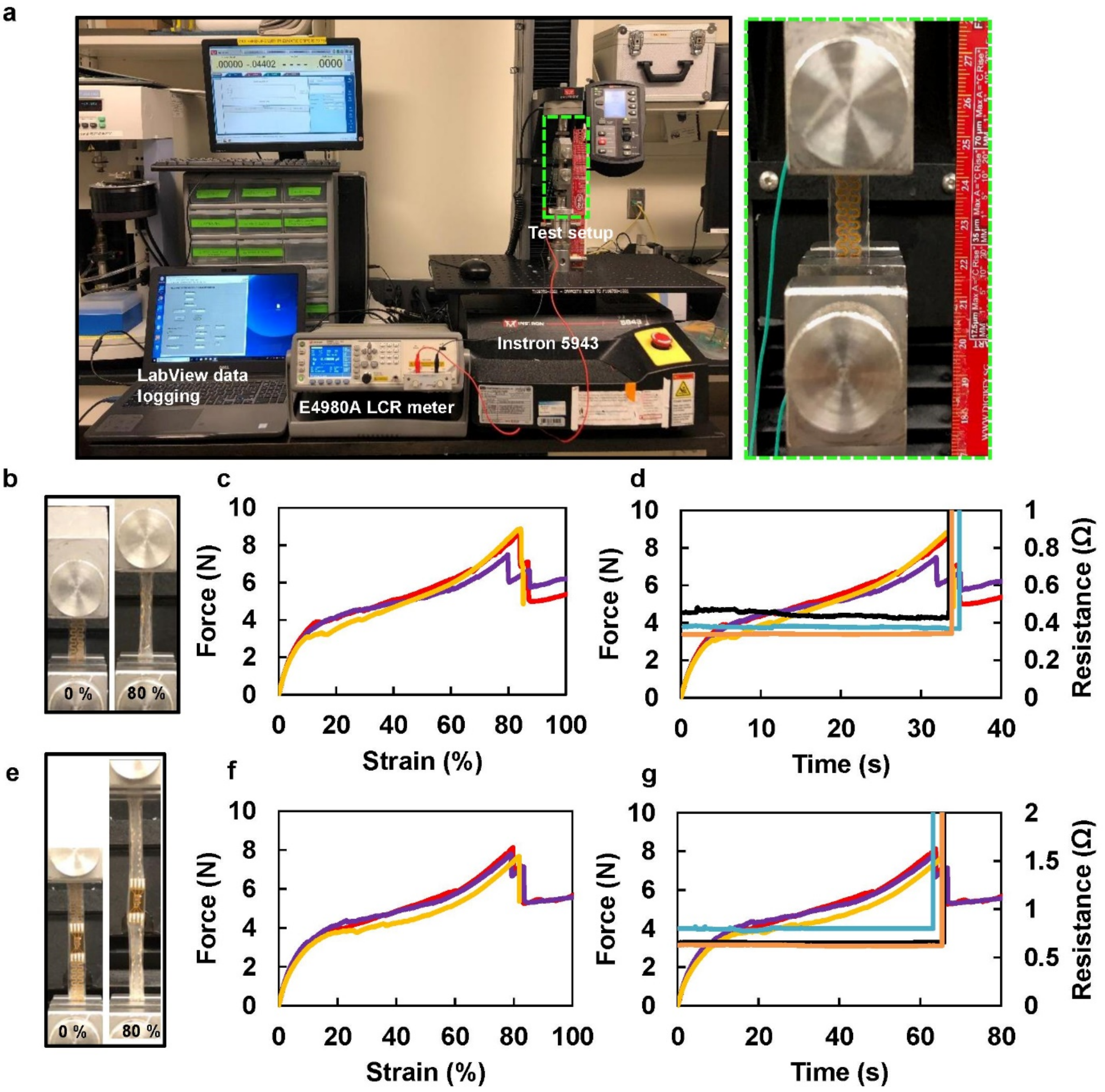

Supplementary Figure 11: Single uniaxial stretching test. a) The photograph of test setup with image of the sample. b) Images of single interconnection at initial and rupture condition. c) Load versus strain measurement graph of three samples in b). d) Transient load and resistance graph of three samples upon stretching. e) Images of sensor island between two interconnects at initial and rupture condition. f) Load versus strain measurement graph of three samples in e), g) Transient load and resistance graph of three samples upon stretching.

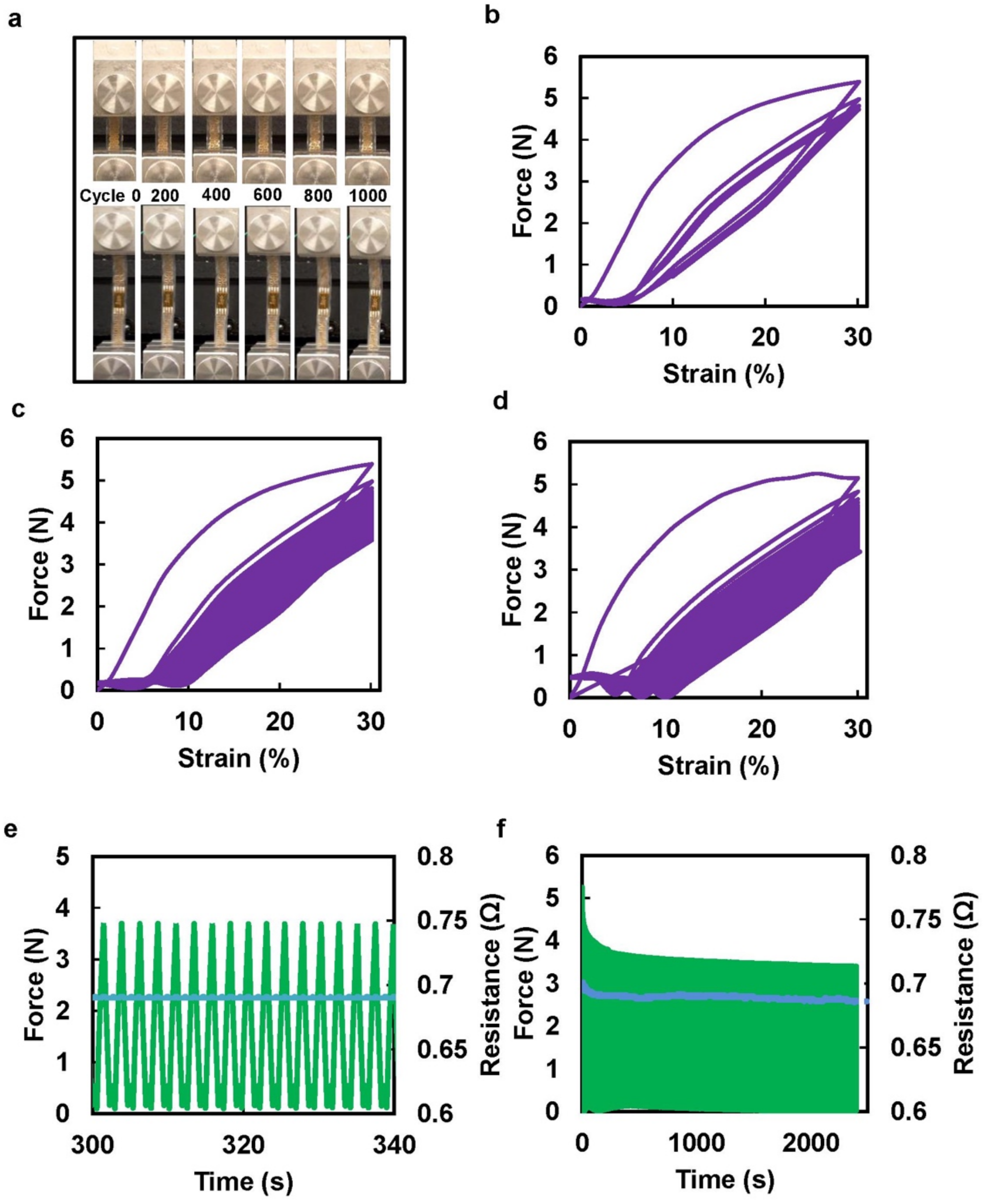

Supplementary Figure 12: Fatigue test of serpentine interconnects. a) Images of the interconnects for every 200 cycles up to 1000 cycles. b) Graph of single serpentine interconnect showing viscoplastic-elastic hysteresis behaviour for the first five repeated strain. c) Continued strain graph until 1000 cycles, d) Graph of a sensor island in between two interconnects showing similar visco-plastic hysteresis behaviour with e,f) a transient response along with resistance value.

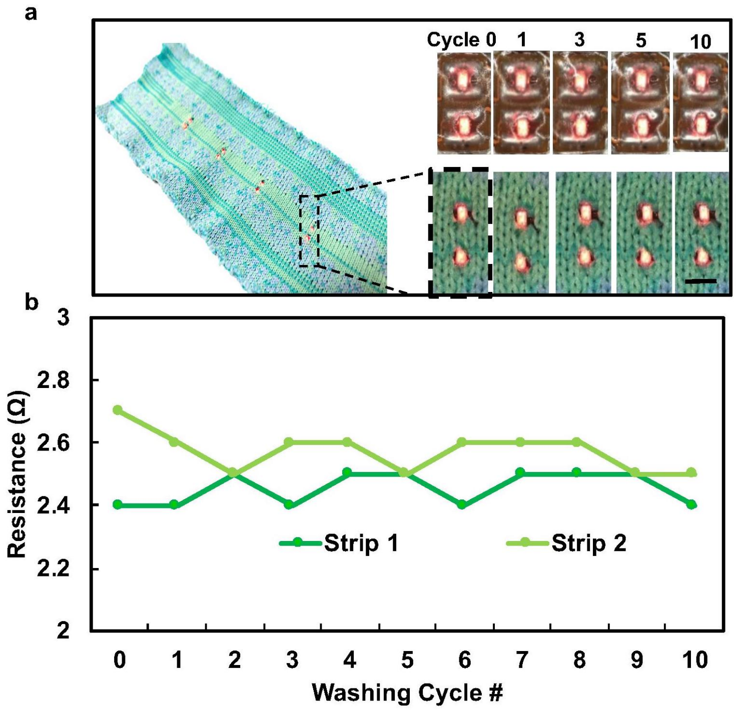

Supplementary Figure 13: Light-emitting diode and interconnect washing test. a) The photographs of LED-embedded fabric throughout 10 washing cycle. Top photographs show the LED strip taken out from the customized fabric while the bottom photographs show the LED strip embedded in the customized fabric (scale bar, 5 mm) and b) Interconnection resistance measurement of the LED strip inside the fabrics throughout 10 cycles of washing, with one a strip containing two groups of four LEDs.

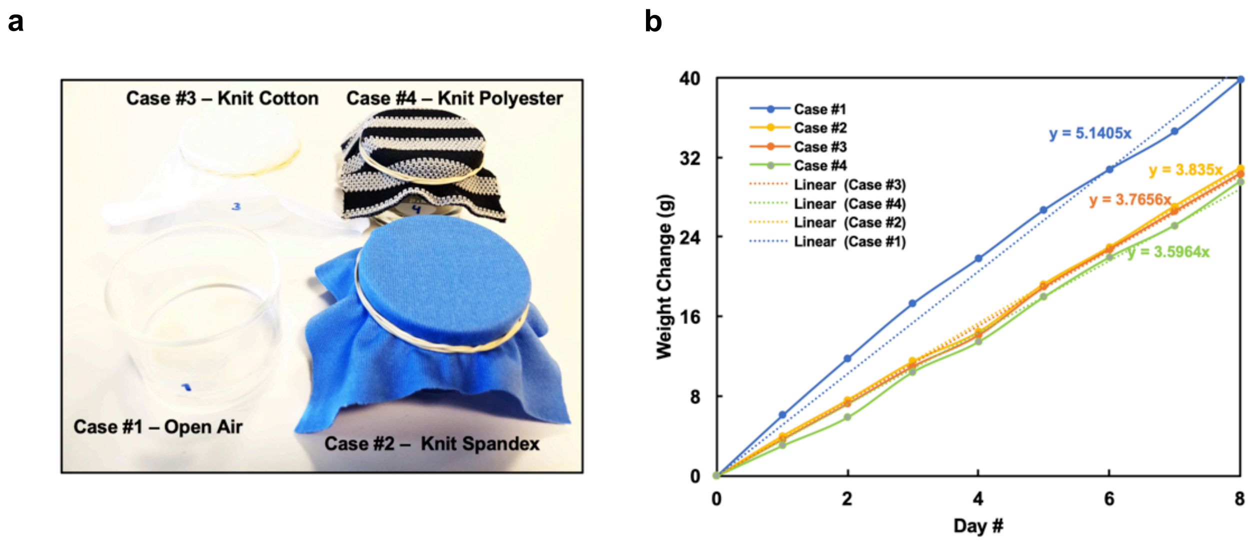

Supplementary Figure 14: Water vapor evaporation rate setup. a) Three different fabrics and an open air (control) under test, b) Accumulated weight change of liquid content in grams per day on every sample.

Case #	WVTR (gm^{-2} /24 hrs)
1 (control)	1335.73
2	996.5
3	978.47
4	934.5

Supplementary Table 5: Calculated water vapor transmission rate for every sample.

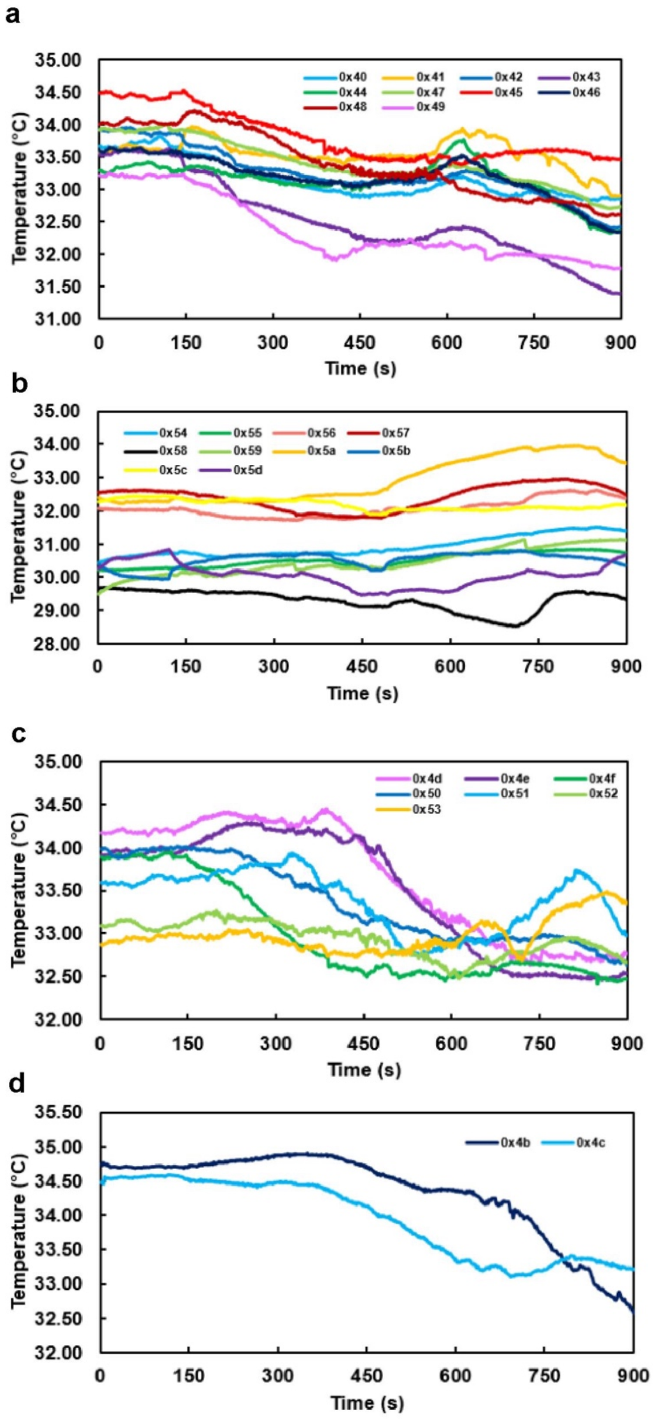

Supplementary Figure 15: Raw uncalibrated temperature sensor graphical data during the 15 minutes test, around the, a) anterior, b) posterior, c) both arms, and d) neck.

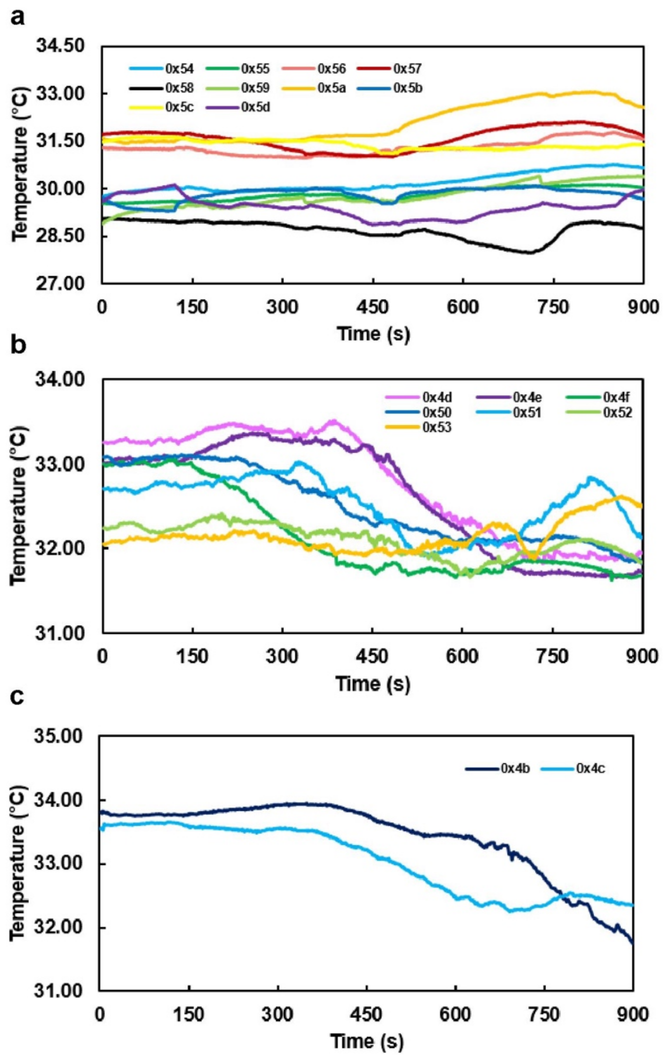

Supplementary Figure 16: Calibrated temperature sensor graphical data during the 15 minutes test around the, a) posterior, b) both arms, and c) neck.

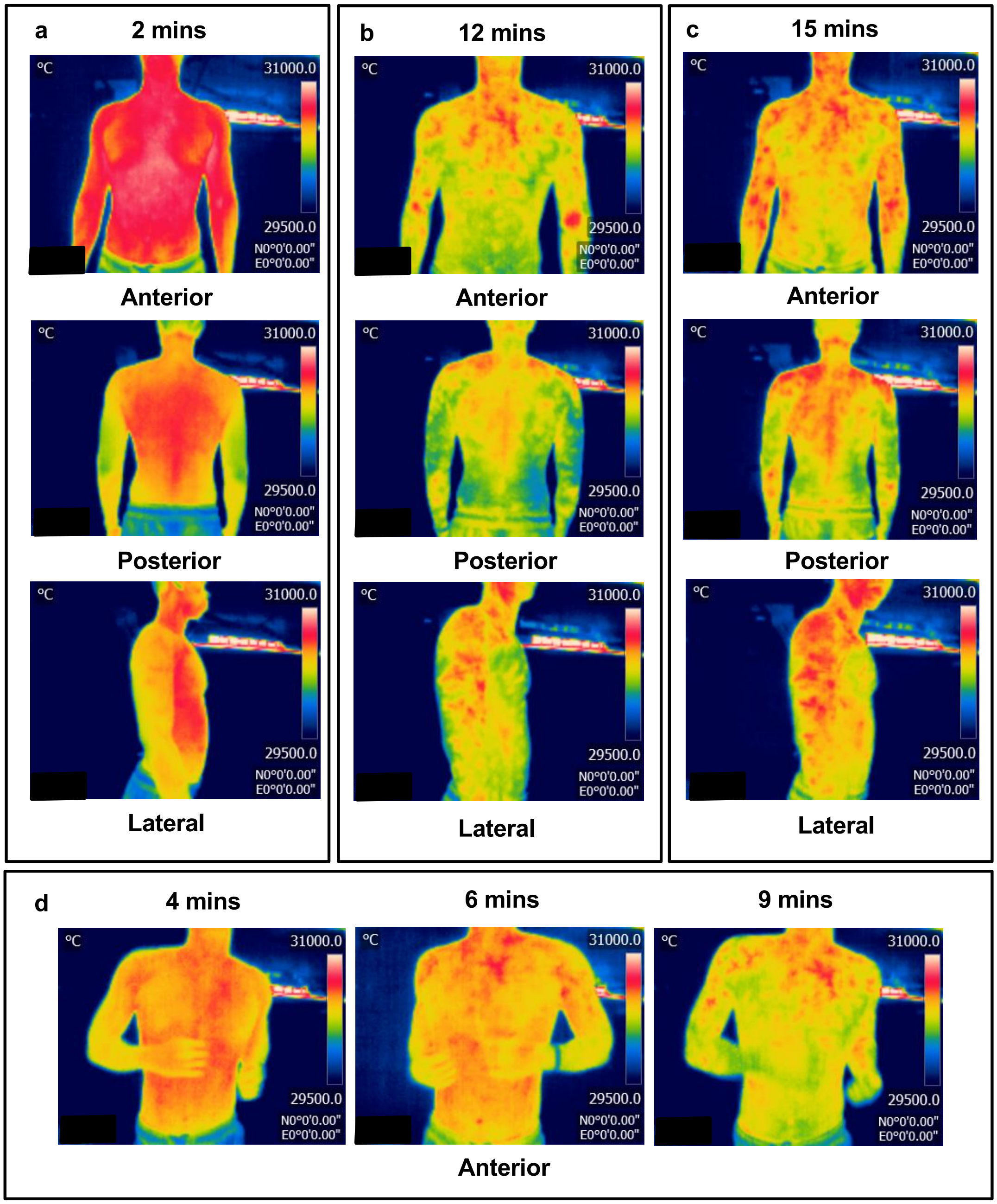

Supplementary Figure 17: Validation images of an IR thermal camera (Duo R, FLIR) with anterior, posterior, and lateral capture throughout the assigned physical exercise running task in a) 2 minutes right before running. b) 12 minutes right after running. c) 15 minutes after 3 minutes of resting, and d) three subsequent times during running.

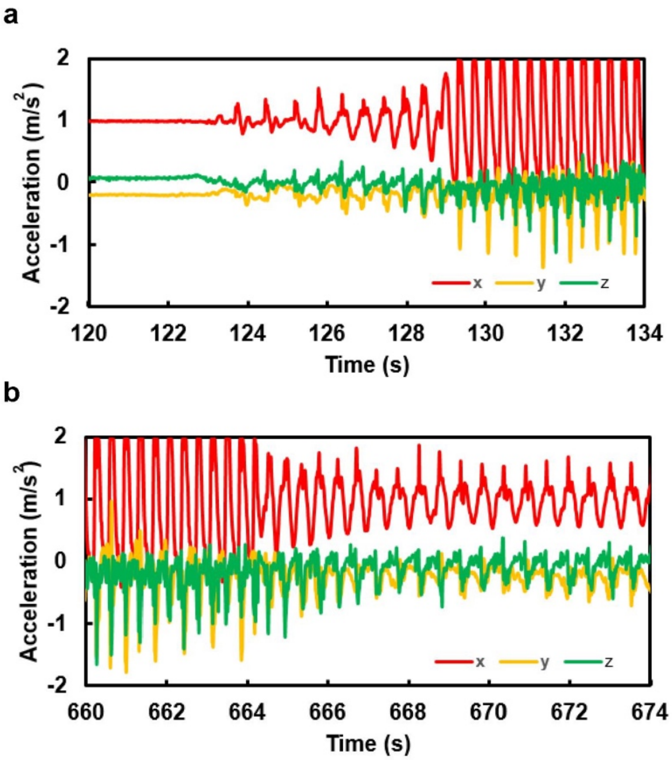

Supplementary Figure 18: Accelerometer graphical data of the subject a) right before running at 6 mph and b) slowing down to 3 mph.

Supplementary References

1. Tanda, G. The use of infrared thermography to detect the skin temperature response to physical activity. in *Journal of Physics: Conference Series* (2015). doi:10.1088/1742-6596/655/1/012062.
2. Han, S. *et al.* Battery-free, wireless sensors for full-body pressure and temperature mapping. *Sci. Transl. Med.* (2018) doi:10.1126/scitranslmed.aan4950.
3. Pantelopoulos, A., Saldivar, E. & Roham, M. A wireless modular multi-modal multi-node patch platform for robust biosignal monitoring. in *Proceedings of the Annual International Conference of the IEEE Engineering in Medicine and Biology Society, EMBS* (2011). doi:10.1109/IEMBS.2011.6091742
4. Park, S. & Jayaraman, S. Adaptive and responsive textile structures (ARTS). in *Smart Fibres, Fabrics and Clothing* (2010). doi:10.1533/9781855737600.226.
